# Hysteresis control of epithelial-mesenchymal transition dynamics conveys a distinct program with enhanced metastatic ability

**DOI:** 10.1038/s41467-018-07538-7

**Published:** 2018-11-27

**Authors:** Toni Celià-Terrassa, Caleb Bastian, Daniel D. Liu, Brian Ell, Nicole M. Aiello, Yong Wei, Jose Zamalloa, Andres M. Blanco, Xiang Hang, Dmitriy Kunisky, Wenyang Li, Elizabeth D. Williams, Herschel Rabitz, Yibin Kang

**Affiliations:** 1 Department of Molecular Biology, Princeton University, Princeton, NJ 08544 USA; 2 Program in Applied and Computational Mathematics, Princeton University, Princeton, NJ 08544 USA; 3 Lewis-Sigler Institute of Integrative Genomics, Princeton University, Princeton, NJ 08544 USA; 4 Department of Mathematics, Princeton University, Princeton, NJ 08544 USA; 5 School of Biomedical Sciences, Queensland University of Technology (QUT), Translational Research Institute, Brisbane, 4102 Australia; 6 Department of Chemistry, Princeton University, Princeton, NJ 08544 USA; 7 Present Address: Cancer Program, IMIM (Hospital del Mar Medical Research Institute), Barcelona, 08003 Spain

## Abstract

Epithelial-mesenchymal transition (EMT) have been extensively characterized in development and cancer, and its dynamics have been modeled as a non-linear process. However, less is known about how such dynamics may affect its biological impact. Here, we use mathematical modeling and experimental analysis of the TGF-β-induced EMT to reveal a non-linear hysteretic response of E-cadherin repression tightly controlled by the strength of the miR-200s/ZEBs negative feedback loop. Hysteretic EMT conveys memory state, ensures rapid and robust cellular response and enables EMT to persist long after withdrawal of stimuli. Importantly, while both hysteretic and non-hysteretic EMT confer similar morphological changes and invasive potential of cancer cells, only hysteretic EMT enhances lung metastatic colonization efficiency. Cells that undergo hysteretic EMT differentially express subsets of stem cell and extracellular matrix related genes with significant clinical prognosis value. These findings illustrate distinct biological impact of EMT depending on the dynamics of the transition.

## Abstract (web summary)

The epithelial-mesenchymal transition (EMT) is a dynamic process that plays important roles in cancer progression and metastasis. Here, the authors characterize a non-linear hysteretic response of E-cadherin repression during TGFβ-induced EMT that is controlled by the strength of the miR-200s/ZEBs negative feedback loop and enhances metastasis.

## Introduction

EMT is a cellular program that occurs in embryonic development, wound healing, fibrosis, and cancer, during which epithelial cells transdifferentiate into a mesenchymal cell fate^1,2^. The conversion involves dramatic phenotypic changes: epithelial cells lose cell polarity and intercellular junctions, rearrange their cytoskeleton, and acquire motile and invasive properties. Importantly, the process is reversible through mesenchymal–epithelial transition (MET), which is essential when migratory cells arrive at their destination to form specific tissues of the embryo^3^. EMT plasticity is also critical during cancer metastasis as it enables tumor cells to acquire the invasive properties necessary to escape the primary tumor and disseminate, extravasate to distant tissues, and subsequently revert back to the epithelial state to form overt metastases and colonize a secondary organ^4,5^. Besides invasion, EMT also endows tumor cells with additional properties, including stem cell-like traits^6^, immune evasion^7^, and chemoresistance^8–10^. However, the requirement of EMT in metastasis has been suggested to be dispensable in some recent studies using genetically modified mouse models^8,9^. It has also been shown that extreme EMT can suppress stem cell properties and reduce metastatic ability if not reverted^11^. Thus, the role of epithelial–mesenchymal plasticity in cancer metastasis is more complicated than initially thought. Notably, many of the previous studies focused on characterizing the endpoint of EMT/MET, while little attention was given to how the cellular dynamics of EMT may have an impact on its metastasis-promoting effect.

The EMT gene program is regulated by a complex network of transcription factors, miRNAs, long non-coding RNAs, epigenetic modulators, and external microenvironmental signals^1,12^. Ultimately, the pathways inducing EMT converge to suppress epithelial genes, such as E-cadherin, which is considered the hallmark molecule of the epithelial status^13^. A potent inducer of EMT is TGF-β, which signals through the TGF-β receptor-Smad pathway to increase the expression of master transcriptional regulators of EMT such as SNAI1 and ZEB1, a zinc-finger transcriptional repressor of E-cadherin^14^. In addition, ZEB1 represses the expression of the miR-200 family of miRNAs, which reciprocally repress ZEB1/2 and TGF-β production^15–19^. The miR-200s/ZEBs negative feedback loop is known to maintain epithelial homeostasis when miR-200 level is high, and it is also the most influential feedback loop for sustaining the mesenchymal state when Zeb1/2 are highly expressed^20,21^. Interestingly, computational studies have indicated non-linear multistable EMT dynamics based on feedback loops at the core of the EMT regulatory network^21–25^, in particular the negative feedback loops between miR-34/SNAI1 and miR-200/ZEB1, which are interconnected bistable switches^24,26^. However, the biological impact of the non-linear EMT dynamics on metastasis remains mostly unknown.

In biological systems, tightly balanced feedback loops produce non-linear responses (switcher mode) and bistability of cellular states, also called hysteresis^27,28^. In this study, we combine mathematical modeling and experimental validation to show that hysteresis control of EMT is critically dependent on the miR-200/ZEB1 double-negative feedback loop. We observe that most, but not all, normal and tumor mammary epithelial cells exhibit hysteretic patterns in TGF-β driven EMT. Hysteresis ensures robust system response to minimal signal in a bidirectional manner, and it is widely observed in different biological regulatory systems^27^. Strikingly, metastatic colonization was only increased in cells undergoing EMT in a non-linear hysteretic mode, in part due to the differential transcriptional regulation of genes, including those involved in stem cell and extracellular matrix (ECM) regulation. Taken together, our study identifies distinct types of EMT dynamics that have functional consequences in metastasis.

## Results

### TGF-β-induced EMT exhibits bistability of E-cadherin levels

**Fig. 1:**
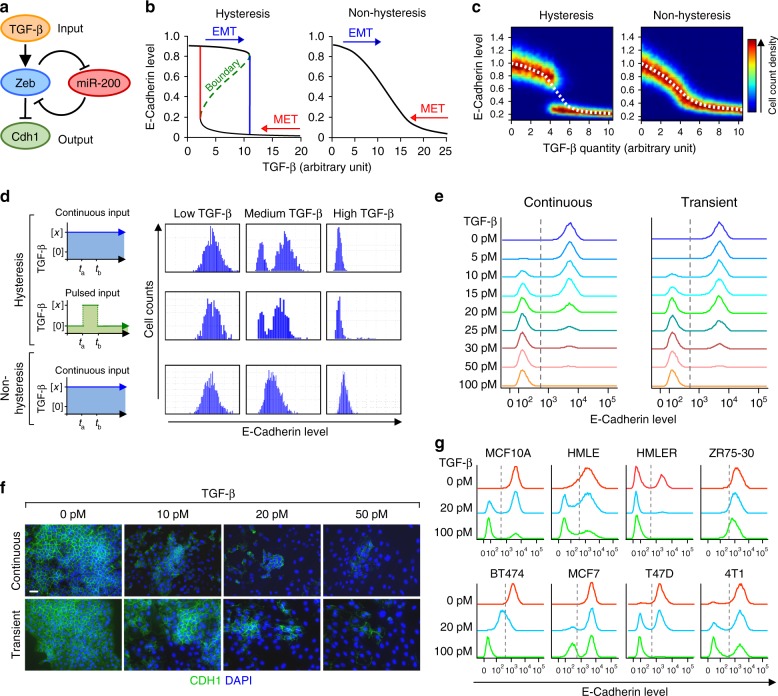
Mathematical modeling and experimental validation of mammary epithelial cells undergoing EMT. **a** Diagram of the simplified model of TGF-β induced EMT and the central role of the miR-200-Zeb double negative feedback loop. **b** Hysteresis within EMT and MET as indicated by CDH1 expression level (left panel) and altered model (non-hysteresis, right panel), based on ODE single cell deterministic model. Within a certain range of TGF-β (2.5-12 arbitrary unit), the steady-state CDH1 level is bistable and is either high or low, depending on the previous state of the cells (i.e. whether it is undergoing EMT or MET). **c** Simulated steady-state calculations of *CDH1* level in a homogeneous collection of cells in increasing dose of TGF-β. Heatmap graphs depict CDH1 expression at single cell level (color indicates cell count for a given CDH1 expression level) and the dashed line represents CDH1 expression as the average of the population. Note the bistable shift in the hysteresis model (left) versus the gradual shift in the non-hysteresis model (right). **d** Computational simulation of CDH1 expression after different regimes of TGF-β treatment in a cell population. **e** Flow cytometry analysis and **f** Immunofluorescence of the endogenous CDH1 expression in parental NMuMG cells after treatment with indicated concentrations of TGF-β for 72 h (histograms, left), or for just 1 h of transient treatment, followed by measurement of CDH1 expression 72 h later (histograms, right). **g** Flow cytometry analysis of CDH1 expression in multiple normal and cancerous mammary epithelial cell lines after 100 pM TGF-β treatment for 9 days. Scale bar: 20 μm in **f**

To interrogate dynamic behavior of gene networks, we derived a mathematical model for TGF-β-induced EMT based on ordinary differential equations (ODE) (Supplementary Mathematical Analysis and Supplementary Tables 2–3). To reduce complexity and control experimental variables, we focused on the most influential components of EMT signaling: TGF-β stimulation (input), miR-200s/ZEBs regulatory axis (intermediate feedback loop), and expression of E-cadherin (output)^20,21,29^ (Fig. 1a). The model is not designed to describe the interconnected modulation of associated genes nor different degrees of EMT states. However, it is configured to characterize the dynamics of the transition from the epithelial state to an EMT-like state, independent of being partial or fully mesenchymal.

The single cell ODE deterministic mathematical model, based on Michaelis–Menten style reaction kinetics and mass transfer, revealed that the steady state E-cadherin expression exhibits pronounced bistability with varying TGF-β stimulation (Fig. 1b, left panel), but not in the altered, non-hysteresis model (Fig. 1b, right panel). In particular, the system possesses two accessible stable steady states of E-cadherin expression (Fig. 1b, left panel, black curves), which are separated by instability of intermediate expression (green curve). The forward orbits of the iterated function system for the ODE system reveal the two steady state attractors, epithelial or mesenchymal (Supplementary Fig. 1a). These suggest E-cadherin expression to experience dynamics of hysteresis control (also known as “bang bang” control). Simulated steady-state dynamics of a collection of homogeneous cells, represented by an ensemble of stochastic ODEs, shows a non-linear transition from high to low E-cadherin levels with increasing TGF-β input (Fig. 1c). Similarly, cell population simulation studies show a bimodal distribution of the transition regardless of whether TGF-β treatment is continuous or transient (pulsed input) (Fig. 1d). However, in a putative system without hysteresis, the transition dynamics are monotone and unimodal, gradually decreasing with increasing TGF-β input (Fig. 1c, d).

We experimentally validated these mathematical predictions using fluorescent-activated cell sorting (FACS) of fixed cells and immunofluoresence (IF) analyses in NMuMG mouse mammary epithelial cells and EpRAS mouse mammary tumor cells, which are classical models to study TGF-β induced EMT^30,31^. High dose (100 pM) of TGF-β treatment induced a shift in the entire population of cells from epithelial state (high E-cadherin) to the mesenchymal state (low E-cadherin) in a 3-day treatment experiment. A bimodal distribution of two distinct populations of cells with high and low level of E-cadherin expression existed within a certain range of TGF-β concentration (particularly between 10–30 pM) (Fig. 1e, f and Supplementary Fig. 1b, c). Interestingly, 1-h transient treatment of TGF-β followed by TGF-βRI kinase inhibitor (LY2109761) to completely block TGF-β signaling was still sufficient to induce similar EMT shift and bimodal transition after 3 days (Fig. 1e, f and Supplementary Fig. 1b, c), indicating that these cells were able to undergo EMT upon a certain threshold of transient TGF-β exposure and have molecular memory. In addition, using a GFP reporter controlled by the *E-cadherin (CDH1)* promoter, we were able to observe the same dynamics of GFP expression, suggesting that the hysteresis control is at the transcriptional level (Supplementary Fig. 1d, e). Finally, to explore the natural existence of different modes of EMT (hysteretic or non-hysteretic), we test the cellular dynamics of EMT in several normal or cancerous mammary epithelial cell lines from human or mouse. Interestingly, while most of the cell lines exhibited hysteresis in TGF-β-induced EMT, the BT474 human breast cancer cell line showed non-hysteretic, unimodal dynamics (Fig. 1g). This result suggests the existence of distinct modes of EMT dynamics in nature.

### Hysteresis is controlled by the miR-200/Zeb1 feedback loop

**Fig. 2:**
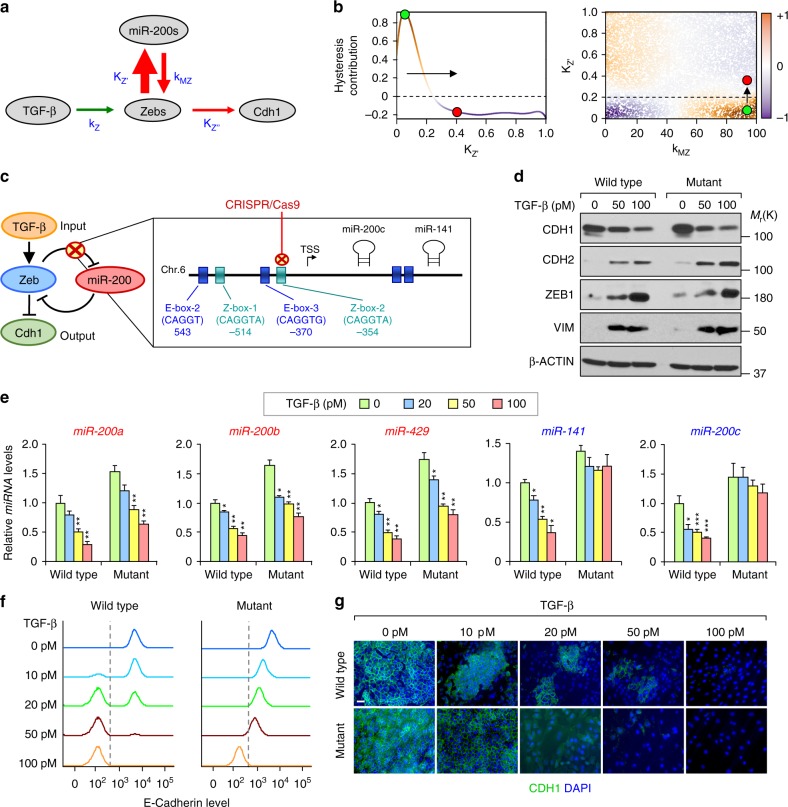
ZEB1 repression of miR-200s tightly controls hysteresis during EMT. **a** Schematic illustration of the strength of interaction constants and reaction coefficients: k_Z_, K_Z′_, K_Z′′ and_ k_MZ._ The thickness of the arrows represents the magnitude of the interaction strength (sensitivity index). **b** High dimensional model representation (HDMR) component functions of hysteresis contribution in K_Z′_ (left panel; first-order) and K_Z′_ vs k_MZ_ (right panel; second-order). The green dot denotes a region with hysteresis (orange background) and red dot a region without hysteresis (blue background). The arrow represents a minimal modification of the K_Z′_ that eliminates hysteresis. **c** Representation of the Z-box-2 deleted using CRISPR-Cas9 in NMuMG and EpRAS cells generating the corresponding mutant cells. **d** Western-blot analysis of EMT markers in wild type and mutant NMuMG clonal cells at the indicated concentration of TGF-β for 72 h. **e** qRT-PCR analysis of the expression levels of, cluster-1 (red) and cluster-2 (blue) miR-200s family members in wild type and mutant NMuMG cells (*n* = 3 biological replicates) treated with the indicated concentrations of TGF-β for 72 h. **f** Flow cytometry analysis and **g** immunofluorescence analysis of CDH1 expression in NMuMG clones after treatment with indicated concentrations of TGF-β for 72 h. Scale bar: 20 μm

To identify the molecular mechanism of hysteresis control in our system of study, we first generated independent random realizations of the model parameters on a scaled hypercube (reflecting maximum entropy). Then, for each random input vector, we estimated a measure of hysteresis extent. The empirical probability density and cumulative distribution functions revealed hysteresis extent to be bimodal, conveying the hysteresis bifurcation (Supplementary Fig. 2a). Then, we used high dimensional model representation (HDMR) to identify the hierarchy of independent and cooperative functional molecular effects, known as HDMR component functions, to elucidate how the model parameters functionally convey hysteresis;^32,33^ the structure of the HDMR component functions additionally conveys a global sensitivity analysis (GSA). From the random input-output data, we estimated first- and second-order HDMR component functions and their sensitivity indices. Through this analysis, we discovered key system parameters to underlie hysteresis: (k_Z_) reaction coefficient of TGF-β induction of Zeb; (K_Z’_) Michaelis–Menten constant of Zeb binding and transcriptional repression of the *miR-200s* promoter; (k_MZ_) reaction coefficient of miR-200s binding and post-transcriptional repression of Zeb; and (K_Z”_) Michaelis–Menten constant of Zeb binding to and transcriptional repression of the *E-cadherin* promoter (Fig. 2a, and Supplementary Table 4). The analyses revealed that the strongest contributor to hysteresis is K_Z’_, suggesting the direct repressive action by Zebs on the *miR-200s* promoter to be critical. Moreover, the K_Z’_ component function is highly non-linear, with significant contribution to hysteresis only occurring for small values of K_Z’_, which correspond to its repressive action ability (Fig. 2b, left panel). Importantly, the second-order HDMR analysis suggests that a small increase on K_Z’_ values eliminates hysteresis of the system by increasing low fidelity repressive action (Fig. 2b, right panel).

To experimentally prove this prediction, we used the CRISPR/Cas9 system to edit the ZEB1 binding sites on the endogenous promoter of miR-200s in NMuMG and EpRAS cells. In particular, we eliminated the Z-box-2 binding site (CAGGTA) on the promoter of cluster-2 miRNAs (miR-200c/miR-141) of the miR-200 family, located on the murine chromosome 6 (Fig. 2c). This binding site is the most conserved Zeb binding site between both miR-200s clusters and has been shown to confer the strongest repressive function^16^. Moreover, it is exclusive for ZEB1 transcription factors; therefore, we were not altering the influence of other transcription factors on miR-200s, such as SNAI1. Overexpression (OE) of ZEB1 did not repress miR-200c levels in the generated mutant cells, unlike the strong suppression of miR-200s by ZEB1 in wild type cells (Supplementary Fig. 2b), indicating that mutation of Z-box-2 was sufficient to impair the negative regulation of ZEB1 of miR-200c/miR-141. As expected, TGF-β treatment reduced cluster-1 (miR-200a/miR-200b/miR-429), but did not reduce cluster-2 (miR-200c/miR-141) levels in mutant cells (Fig. 2e), and consequently the increase of *Zeb1/2* expression levels was slightly weaker compared to wild type cells (Supplementary Fig. 2c). Likewise, in BT474 cells that did not display hysteresis (Fig. 1g), there is a lack of miR-200c down-regulation upon ZEB1-OE and TGF-β treatment (Supplementary Fig. 2d). Importantly, despite the mentioned changes and slightly higher basal levels of miR-200s in mutant cells (Fig. 2e), we observed that NMuMG and EpRAS mutant cells still underwent EMT upon continuous TGF-β induction to a similar degree as their wild type counterparts based on analysis of EMT marker expression (Fig. 2d and Supplementary Fig. 2e). This result suggests that our precise genomic manipulation of the *miR-200c* promoter can subtly alter the natural balance of the miR-200s/ZEBs axis without preventing cells from undergoing EMT. It is also important to note that in this model of EMT induced by 100 pM TGF-β, none of the cells reached an extreme EMT state, as seen by some residual expression of E-cadherin shown by western blot analysis (Fig. 2d and Supplementary Fig. 2e).

Finally, we analyzed the EMT dynamics by monitoring E-cadherin expression of different wild type and mutant clonal populations using FACS and IF. In response to treatment using increasing concentrations of TGF-β, both NMUMG-ΔZ-box-2 and EpRAS-ΔZ-box-2 (hereafter referred as mutant cells) showed a unimodal gradual transition to a mesenchymal state (Fig. 2f, g and Supplementary Fig. 2f), in contrast to the bimodal transitions observed in their wild type counterpart. We consistently observed the same phenomenon in multiple wild type and mutant clones of NMuMG cells (Supplementary Fig. 2g), indicating that the change in EMT dynamics is due to the specific Z-box mutation rather than clonal variations. Therefore, disruption of the Zeb/miR-200 feedback loop led to the lost of bistability (hysteresis) in EMT, and turned it into a gradual linear transition (non-hysteresis). These results confirmed the predictions by HDMR and demonstrated that a modest disturbance of the miR-200s/ZEB1 double-negative feedback loop disrupts the hysteresis bifurcation and dramatically alters the cellular dynamics of EMT.

### Hysteresis sets the threshold and persistence of EMT

**Fig. 3:**
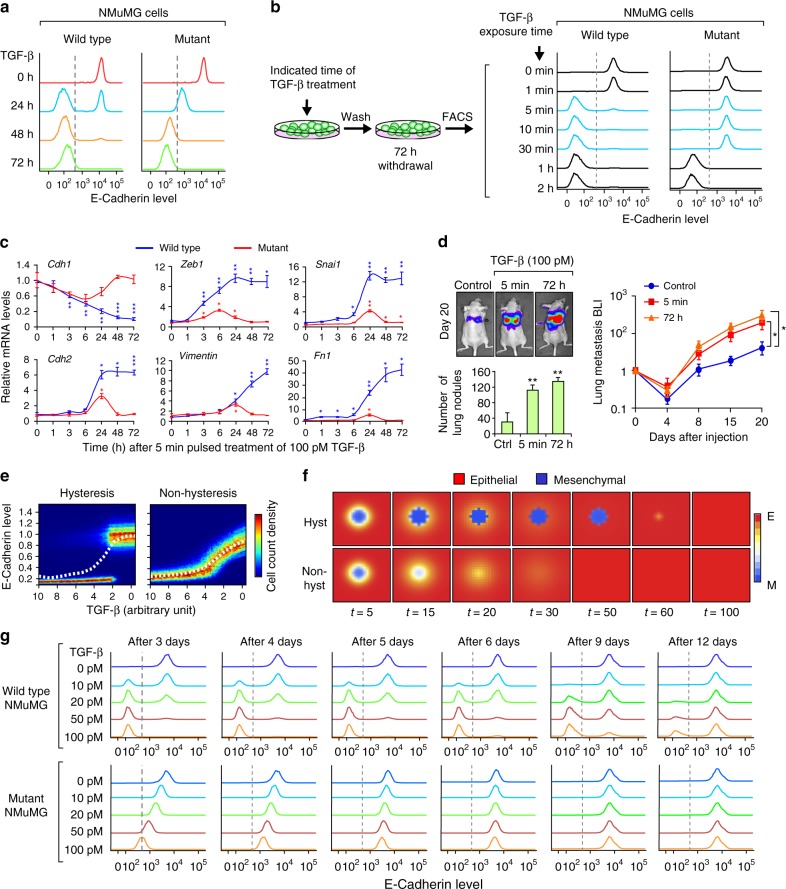
Dynamic analysis of the hysteretic and non-hysteretic EMT in NMuMG and EpRAS cells. **a** Analysis of the dynamic of EMT induction (100 pM TGF-β) in wild type and mutant NMuMG cells by flow cytometry analysis of CDH1. The time represents the duration of treatment at the moment of measurement. **b** Minimum exposure time of 100 pM TGF-β required to induce EMT in wild type and mutant NMuMG cells by flow cytometry analysis of CDH1. The time represents the duration of the transient treatment, with the measurement done 72 h post-induction for all conditions. **c** qRT-PCR analysis of EMT marker genes at the indicated times after a 5 min pulse treatment of 100 pM TGF-β. *n* = 3 technical replicates. **d** Quantification of lung metastatic nodules and bioluminescence imaging (BLI) quantification of the metastatic growth of wild type EpRAS cells in the lungs after tail vein (T.V.) injection of 20,000 cells in Ncr-nu/nu mice (*n* = 6 mice). Cells were untreated (control) or treated with 100 pM TGF-β for the indicated time. **e** Steady-state simulation of CDH1 expression in mesenchymal-like cells after withdrawal of TGF-β. Heatmap graphs depict CDH1 expression at single cell level (color indicates cell count for a given CDH1 expression level) and dashed line represents CDH1 expression as the average of the population. **f** Simulation of EMT reversion in mesenchymal-like cell populations by modeling CDH1 expression with or without hysteresis. Red represents the epithelial cells (CDH1-high) and blue the mesenchymal cells (CDH1-low). **g** Flow cytometry analysis of the CDH1 expression showing the reversion kinetics in wild type versus mutant NMuMG cells. Cells were treated with indicated concentration of TGF-β for 72 h, after which TGF-β was withdrawn and cells allowed to revert for indicated duration. Data represent mean±SEM in **c** and **d**. *P < 0.05, **P < 0.01, ***P < 0.005 by two-tailed Student’s *t*-test in **c** and **d**

To further analyze the temporal kinetic differences of hysteretic and non-hysteretic EMT, which might have relevant physiological importance, we characterized different induction times and steady states using the wild type (hysteresis) and mutant (non-hysteresis) cells. While both wild type and mutant NMuMG cells switched off E-cadherin expression after 2 days of continuous TGF-β (100 pM) treatment, more than 60% of wild type population had already completed the transition after 24 h (Fig. 3a). Instead, the mutant cells still displayed medium E-cadherin levels at 24 h. We next interrogated the minimal exposure time of TGF-β required to induce EMT after 3 days of treatment. Interestingly, a 5-min pulse of TGF-β treatment was enough to induce EMT in the entire population of wild type cells, while non-hysteretic mutant cells required at least 1 h of TGF-β treatment to undergo EMT, as measured by FACS of E-cadherin (Fig. 3b). We did not observe significant difference in SMAD2/3 phosphorylation between wild type and mutant cells upon TGF-β treatment (Supplementary Fig. 3a) despite a slight decrease of endogenous *TGF-β1* mRNA levels in mutant cells (Supplementary Fig. 3b), suggesting that the difference in EMT is unlikely to be due to variation in TGF-β signaling strength. In agreement with the FACS result (Fig. 3b), the time course analysis of EMT marker genes demonstrated that only hysteresis EMT in wild type cells can engage the EMT gene program with only 5-min short pulse of TGF-β treatment (Fig. 3c). Of note, *Zeb1* induction and *Cdh1* downregulation preceded *Snai1* induction and other changes of EMT markers (*Cdh2*, *Vim*, *Fn1*), suggesting Zeb1 as the initial driving factor of hysteretic EMT. In contrast, such EMT-related gene expression changes are lacking or at least are not sustained in mutant cells. Consistently, while continuous TGF-β treatment was able to suppress cluster-1 miR-200 (such as miR-200b) expression in mutant cells, 5 min pulsed TGF-β treatment was not able to do so (Supplementary Fig. 3c). These differences highlight the importance of hysteresis in EMT response to short term exposure to TGF-β. Importantly, 5 min short pulse of TGF-β treatment in wild type EpRAS cells was enough to increase lung metastasis similar to that of the continuous TGF-β treatment (Fig. 3d), which highlights the functional and physiological importance of the EMT dynamics.

Our mathematical model predicted a bidirectional effect of EMT-MET hysteresis as reflected by the re-acquisition of E-cadherin expression with the removal of TGF-β (Fig. 3e). To examine the spatiotemporal dynamics of hysteresis vs non-hysteresis EMT, we mathematically modeled the persistence of EMT and MET reversion to the epithelial state by incorporating autocrine dynamics into the core model and by equipping the TGF-β field with diffusion, organizing cells into a 2D periodic array (Fig. 3e). We simulated and predicted that a cell population in the mesenchymal-like state has a time-extended mesenchymal persistence by hysteresis, whereas without hysteresis the system displays an earlier gradual reversion (Fig. 3f and Supplementary Table 5). To experimentally validate the mathematical prediction of hysteresis during MET, we first treated the NMuMG and EpRAS cells for 3 days with different concentrations of TGF-β to reach the mesenchymal state in both wild type and mutant cells, and then withdrew TGF-β and measured E-cadherin by FACS in the following days. Consistent with mathematical prediction, E-cadherin kinetics showed that the wild type cells followed bimodal reversion which was completed after 12-15 days, whereas the mutant cells reversed to the epithelial phenotype in a linear gradual mode within 4–5 days after TGF-β withdrawal (Fig. 3g and Supplementary Fig. 3d, e). This result showed that MET also follows hysteresis and maintains extended mesenchymal state in wild type cells, while the mutant cells follow a linear and accelerated MET.

### miR-200/Zeb1 balance primes the spread of EMT

**Fig. 4:**
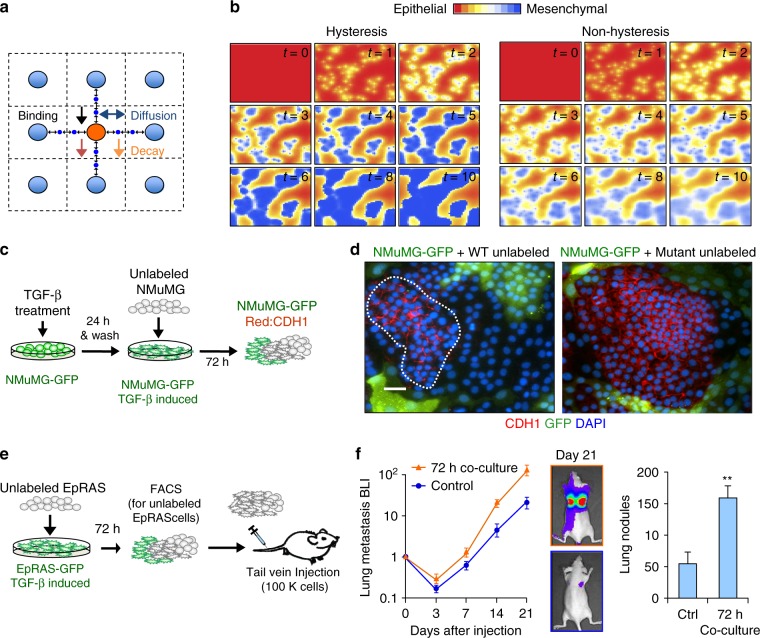
Hysteresis fosters the EMT neighborhood spreading effect. **a** Euclidean stencil grid representing the parameters considered for the neighborhood effect computation. **b** Computational spatial simulation of the EMT spreading paracrine effect with and without hysteresis in a population of cells at different time points. **c** Schematic representation of the co-culture treatment process. NMuMG-GFP cells were induced with 100 pM TGF-β and for 24 h, after which unlabeled NMuMG cells were added into the culture. Immunofluorescence analysis was performed 72 h later. **d** Immunofluorescent images showing loss of CDH1 expression (red) in GFP-positive cells that were pre-treated with TGF-β to undergo EMT. Among GFP-negative wild type cells that were not pre-exposed to TGF-β some of the them lost CDH1 expression under the influence of neighboring GFP-positive, mesenchymal-like cells, while the rest (highlighted by dashed line) remained epithelial (left panel). Most of GFP-negative mutant were not able to undergo EMT through the neighborhood effect (right panel). Scale bar: 20 μm. **e** Schematic representation of co-culture procedure of EpRAS cells (left). EpRAS-GFP cells were induced with 100 pM TGF-β and for 24 h, after which unlabeled EpRAS cells were added into the culture. After 72 h of co-culture, GFP-positive and -negative cells were separated by flow cytometry. 100,000 unlabeled EpRAS cells with or without the co-culture treatment were injected by tail vein injection into Ncr-nu/nu mice (*n* = 6 mice). **f** Left: bioluminescence imaging (BLI) quantification and representative images of the metastatic growth of EpRAS cells. Right: quantification of lung metastasis nodules (*n* = 6 mice). Data represent mean±SEM. ** *P* < 0.01 by two-tailed Student’s *t*-test

Previous studies have reported that EMT-like cells secrete TGF-β and sustain EMT in an autocrine/paracrine fashion through the miR-200s/ZEBs signaling axis^20^. We simulated treatment of random cells as point sources of TGF-β signaling, enabling a paracrine regulatory spreading effect shown in the Euclidean stencil grid (Fig. 4a). In the computational simulation, EMT spreads across the tissue in a patch-forming manner with random edges following from diffusion of TGF-β (Fig. 4b and Supplementary Table 6), similar to what we observed in cultured cells undergoing TGF-β-induced EMT (Fig. 1f). In computational simulation of non-hysteresis conditions, the paracrine TGF-β spreading effect was weakened with mild gradual decrease of E-cadherin (Fig. 4b and Supplementary Table 6). This is consistent with the lack of molecular memory of linear systems and the notion that the mesenchymal state is buffered by the autocrine induction of the miR-200s/ZEBs axis. To experimentally prove these predictions, we performed cell co-culture assays. We first induced EMT in NMuMG-GFP cells by TGF-β treatment. 24 h later, unlabeled cells were added for co-culture and analyzed 72 h later by immunofluorescence analysis of CDH1. EMT-induced NMuMG-GFP cells instigated EMT in untreated NMuMG cells, leaving behind few patches of epithelial cells, whereas the mutant cultures were not able to spread the signal efficiently and only mild inductions are observed in a diffusive fashion (Fig. 4c, d, right panel and Supplementary Fig. 4b). Notable, several TGF-β-induced pro-metastatic genes, such as *Angptl4*, *Jag1, Mmp9*, and *Vegfa* were induced in the wild type NMuMG cells, likely by the paracrine TGF-β spreading effect during co-culture, but not in the mutants due to their inability to undergo EMT by the spread effect (Supplementary Fig. 4c). Accordingly, untreated wild type EpRAS cells had significant increase in lung metastasis ability in vivo when they are in co-cultured with EMT-like cells (Fig. 4e, f). Overall, these results demonstrate the impact of hysteresis EMT at the populational level, with functional importance in promoting metastatic competency of neighboring cells.

### Non-linear hysteresis EMT dynamics increases metastasis

**Fig. 5:**
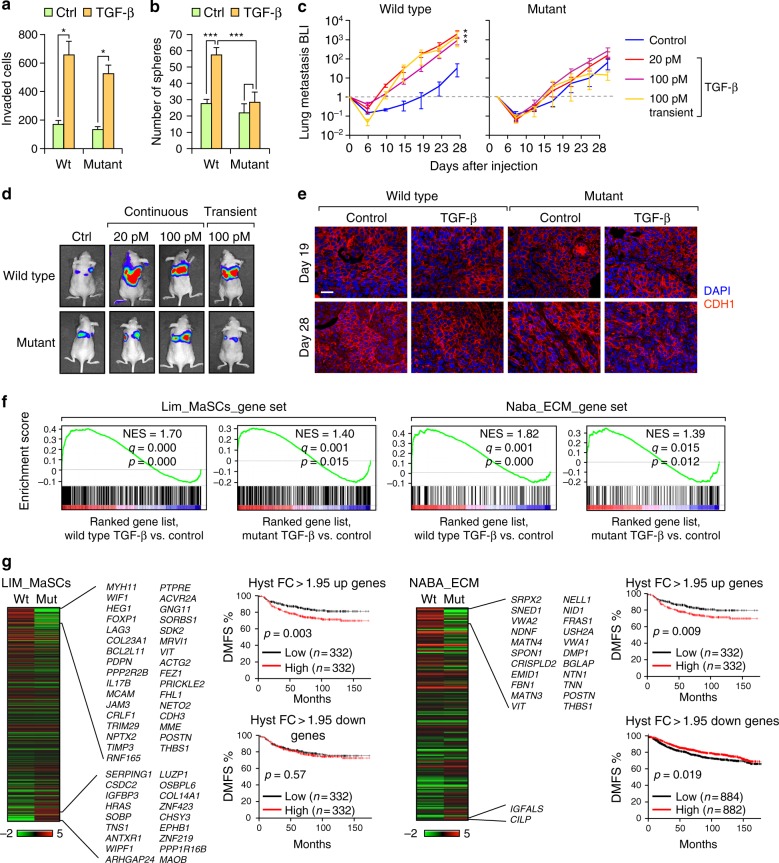
Hysteresis during EMT increases the metastatic colonization ability of EpRAS cells. **a** Quantification of invading cells in Matrigel transwell invasion assays after 24 h of seeding 100,000 EpRAS cells with or without 100 pM TGF-β treatment. *n* = 3 biological replicates; data represents mean±SEM. **b** Quantification of mammosphere formation of 5,000 EpRAS cells with or without 100 pM TGF-β treatment. *n* = 4 biological replicates; data represents mean±SEM). **c**, **d** Bioluminescence imaging (BLI) quantification (**c**) and representative images (**d**) of the metastatic growth of the EpRAS wild type and mutant cells in the lungs after tail vein injection of 20,000 cells in Ncr-nu/nu mice (*n* = 6 mice). EpRAS cells were treated with the indicated concentration of TGF-β for 72 h (continuous) or 5 min followed by 72 h withdrawal (transient) prior to injection. Data represents mean±SEM. **e** Confocal microscopy imaging of CDH1 staining of lung metastatic tumor tissue. Wild type and mutant EpRAS cells were injected at the indicated conditions. Lung metastasis sample collection was done at the indicate time points after tail vein injection. Scale bar: 25 μm. **f** GSEA demonstrating the enrichment of the gene sets from the MSigDB related to mammary stem cells (Lim_MaSC_M2573)^50^) and ECM (Naba_ECM-GLYCOPROTEINS_M3008) in the ranked gene list of TGF-β treated vs. control conditions in wild type or mutant EpRAS cells. NES, normalized enrichment score. **g** Heatmap of differentially expressed genes in Lim_MaSC (left) and Naba_ECM-GLYCOPROTEINS datasets (right); color legend represents Log2 fold change (FC). Genes highlighted are genes with a >1.95 fold change (FC) during EMT in wild type but are not significantly changed in during EMT in mutant EpRAS cells. Kaplan–Meier distant metastasis free survival (DMSF) curves of breast cancer patients in the composite KM plotter breast cancer clinical database^36^ with higher or lower than median expression levels of the indicated gene sets were shown in the right side of the heatmap. *P < 0.05, **P < 0.01, ***P < 0.005 by two-tailed Student’s *t*-test in **a**–**c**. *P*-value by log-rank tests in **f**

We next investigated whether the different modes of EMT (hysteretic vs non-hysteretic) have any impact on the functional properties of the cells. In vitro invasion assays showed a similar gain of invasive ability in both wild type and mutant EpRAS and NMuMG cells after TGF-β induced EMT (Fig. 5a and Supplementary Fig. 5a), which was expected based on the similar degree of EMT markers expression changes (Fig. 2d and Supplementary Fig. 2e) and cell morphology (Supplementary Fig. 5b). In contrast, tumorsphere formation assays in vitro showed increased sphere-forming ability in wild type cells undergoing hysteretic EMT but not in mutant cells (Fig. 5b). In vivo mammary fat pad limiting-dilution injections demonstrated a significant increase in tumor-initiating cell (TIC) frequency in TGF-β-induced wild type EpRAS cells compared to mutant cells (Supplementary Fig. 5c). We next performed tail vein injections to test lung metastatic colonization. Importantly, wild type EpRAS cells, but not mutant cells, had higher ability to form lung metastasis than mutant cells after TGF-β-induced EMT (Fig. 5c, d). Immunofluorescence and confocal microscopy analysis of the metastatic tissues after 19 and 28 days showed the same degree of tumor re-epithelization of E-cadherin expression in both systems over the time, including non-treated control cells (Fig. 5e), indicating that the increase in metastasis was not due to extended maintenance of the EMT-like phenotype during metastatic formation. Overall, these results demonstrated that hysteretic EMT induces tumor initiation and pro-metastatic properties much more prominently than non-hysteretic EMT, highlighting the previously unknown importance of the EMT dynamics in the pathological behavior of cancer cells during metastasis initiation.

Based on these results, we hypothesized that changes in the dynamics of EMT may cause an alteration of the associated EMT gene programs and cellular features related to metastatic ability. Therefore, we performed mRNA transcriptomic profiling, comparing wild type and mutant EpRAS cells after 3 days of 100 pM TGF-β treatment when all cells have reached EMT in both wild type and mutant EpRAS cells. Gene set enrichment analysis (GSEA) revealed a high number of genes differentially expressed between wild type and mutant EpRAS cells after TGF-β-induced  EMT. A total of 2413 genes were differentially expressed (FC > 2) among the wild type and mutant cells. Despite no major changes in the degree of enrichment of the EMT_HALLMARK genes between wild type and mutant cells (Supplementary Fig. 5d), as expected, a subset of 22 miR-200 target genes were uniquely upregulated in the TGF-β treated wild type cells and not in mutant cells (Supplementary Fig. 5e), presumably due to the inability of TGF-β treatment to decrease cluster-2 miR-200 levels in the mutant cells (Fig. 2e). Interestingly, the mammary stem cell gene set (Lim_MaSCs) and the ECM (Naba_ECM_GLYCOPROTEINS) were more strongly enriched by the hysteretic EMT (Fig. 5f), with 33 and 22 genes upregulated by >1.95 fold by hysteretic-EMT, respectively (Fig. 5f). Many of these genes are related to stem cells properties and ECM proteins, which are two important features associated with increased metastatic colonization^34,35^. Importantly, each subset of differentially expressed genes was linked to poor-prognosis in metastasis-free survival when analyzed in the KM plotter breast cancer clinical database^36^ (Fig. 5g). On the other hand, ECM genes specifically down-regulated by hysteretic EMT (FC > 1.95) is linked to good prognosis while MaSC-related genes down-regulated in hysteretic EMT did not show prognosis power. These results suggested a pro-metastatic gene expression program exclusively acquired by hysteretic EMT.

## Discussion

EMT has long been linked to cancer progression and metastasis. However, there are still ongoing debates regarding its functional relevance in metastasis as different conclusions were drawn based on different model systems used^8,9,37,38^. In our current study, we characterize two types of EMT dynamics (hysteretic and non-hysteretic) that notably influence the metastatic ability of the cancer cells, revealing an additional aspect to consider when studying EMT in cancer metastasis.

Previous computational studies have reported multistability of EMT controlled by the Snai1/miR-34 and Zebs/miR-200s feedback loops^21,23,25^. In addition, TGF-β has been shown to induce a bistable EMT in MCF10A cells^24^. While Tian et al. suggests that the miR-34/SNAI1 and miR-200/ZEB1 loops function as bistable switches^23^, Lu et al. suggested that the multistability predictions are due to the miR-200/ZEB1 axis^21,22^, with the miR-34/SNAI1 axis functioning as a noise-buffer integrator of the system^21^. Using CRISPR/Cas9-based manipulation of Zeb1 binding and repression of *miR-200c/miR-141* promoter activity, we identified the tightly balanced miR-200s/Zeb1 feedback loop as the key hysteresis controller of EMT. This is in agreement with previous experimental and computational studies suggesting the Zeb/miR-200s axis as the key regulatory loop in EMT induction^20,21^. Additionally, despite the reciprocal regulation of miR-200s and Snai1^24,26^, our short-pulse TGF-β treatment data suggests Zeb1 and miR-200s as the main hysteresis controllers. However, we cannot rule out an additive or enhancing effect of latter players, such as Snai1, in enforcing the phenotypic state. In addition, other cell types may have higher dependency on Snai1, instead of Zeb1, depending on the cellular context. Moreover, we found that not all cellular systems exhibit hysteretic EMT dynamics, suggesting that these cell lines may have a disrupted equilibrium of the miR-200s/ZEBs feedback loop. For instance, mutations and epigenetic alterations on the promoters of the critical players, or the status of interconnected pathways, such as p53-dependent regulation of miR-200s^39^, could disrupt the miR-200s/ZEBs equilibrium. Therefore, both EMT dynamics (hysteretic and non-hysteretic) exists in nature and are likely to dependent on the cellular context and cell of origin.

The EMT dynamics might have important implications in physiological settings. An important finding from our study is the high sensitivity and memory of a hysteretic EMT: a short transient (5 min) exposure of picomolar quantities of TGF-β is sufficient to dictate the phenotypic EMT state of the cells for days, and is sufficient to increase metastasis. However, the absence of hysteresis reduces transition speed and steady-state resilience, and requires longer duration of TGF-β to induce and maintain EMT. Therefore, for tumor cells capable of undergoing hysteretic EMT, a short duration of exposure to TGF-β produced by tumor stromal cells, such as carcinoma associated fibroblasts (CAFs) or platelets, during invasion and dissemination may be sufficient to commit the disseminated tumor cells (DTCs) to EMT, and facilitate extravasation and early seeding of cancer cells at distant organs to promote metastasis formation. Hysteretic EMT therefore enables the transition to mesenchymal-like state without the need for long exposure of TGF-β, which have been shown to restrict cellular plasticity and induce cell differentiation as well as tumor suppression^40,41^. As cellular plasticity is considered as a core condition for tumor progression and metastasis^42–44^, hysteretic EMT may provide the necessary flexibility for cancer cells and avoid extreme phenotypes while subtle change in the miR-200-Zeb double negative feedback loop resulted in a more rigid non-hysteretic system.

Previous studies have reported the existence of distinct EMT programs depending on the specific EMT-inducing transcription factors^45,46^ and the cellular context^43^. In our study, although both types of transition (hysteresis or non-hysteresis) reached the same degree of mesenchymal marker expression, hysteretic EMT cells expressed different subsets of genes related to metastasis and poor clinical outcome. This suggests the existence of distinct EMT programs and the critical role of the dynamics in influencing the downstream cascade of EMT-associated genes. In fact, it has been previously reported that the particulars of the timing of transition and the sequence of the EMT/MET events are essential for acquiring the pluripotent state, in normal somatic cells^47^. Indeed, EMT program is subjected to a very complex regulatory cascade leading to EMT and is associated with diverse downstream gene programs^1^. Therefore, it is conceivable that the end phenotypic state is sensitive to the order and dynamics of key molecular events in EMT. In our study, we demonstrated how a hysteresis controller influences key genes related to the extracellular matrix (ECM) and stemness, both of which play important roles in regulating metastasis^35^. The ECM allows DTCs to recreate their own microenvironment and supportive niche, which potentiates the self-renewal (stemness) activity of DTCs to initiate metastasis. In particular, genes such as periostin (POSTN), which is differentially upregulated with hysteresis (Fig. 5g) has been reported to be critical for lung metastasis and stemness^34^. The increase of metastasis by hysteretic EMT may be due to the combination of higher sensitivity and longer persistence of EMT, as well as the subset of tumorigenic and pro-metastatic genes specially enhanced by hysteretic EMT. In addition, the reduced basal TGF-β level in non-hysteretic mutant cells may also contribute to their inability to respond to TGF-β induced EMT with increased metastatic potential. Consistently with our experimental observation in animal models, the subsets of genes specifically enhanced by hysteretic EMT correlate with poor prognosis. Taken together, our study reveals how different dynamics of EMT have different functional metastatic consequences and clinical prognosis outcomes.

## Methods

### Animal studies and bioluminescence analysis

All procedures involving mice and experimental protocols were approved by the Institutional Animal Care and Use Committee (IACUC) of Princeton University. For limiting dilution assays to test tumor initiating cell (TIC) activity, we performed orthotopic mammary tumor injection of EpRAS in serial dilution numbers into the mammary fat pad (MFP) of female Balb/c mice. Six glands were used for each experimental group and the primary tumors were monitored weekly by palpation. Tumor monitoring and measurement were performed by trained technicians in a blinded fashion. Tumors were considered established when they became palpable for 2 consecutive weeks. ELDA (Extreme limiting dilution analysis) software was used to calculate the frequency of TICs with 95% of confidence. For lung metastasis colonization studies, the indicated number of F-luciferase-labeled EpRAS cells was injected into the lateral tail vein of female athymic Ncr-nu/nu mice. Development of metastases was monitored by bioluminescent imaging (BLI) following retro-orbital injection of 75 mg/kg d-Luciferin. Randomization among litters was performed before injection, and animals were of similar age (6–8 weeks). No statistical method was used to predetermine the sample size. Bioluminescence images were acquired with a Xenogen IVIS 200 Imaging System and analysis was performed using Living Image software by measuring photon flux in the lungs of mice. Data were normalized to the baseline signal on day 0.

### Cell lines, culture conditions and treatments

Cell lines used in this study, including NMuMG (mouse mammary epithelial cell line), HMLE and MCF10A (human mammary epithelial cell lines), EpRAS and 4T1 (mouse mammary tumor cell lines), and human breast cancer cell lines HMLE-R, MCF7, T47D, ZR75-30, and BT474, were cultured using the standard conditions according the American Type Culture Collection (ATCC) instructions. All cell lines were verified negative for mycoplasma contamination by monthly PCR analysis. No cells lines used here appear in the database of commonly misidentified cell lines (ICLAC). All cell lines were validated with STR analysis and compared to NCBI repository data. GFP/F-luc labeled EpRAS cells were transduced with a lentiviral vector expressing both GFP and firefly luciferase proteins. For EMT induction assays, recombinant TGF-β1 (R&D Systems) was diluted in PBS and added to cultured cells at the indicated concentration. Different treatment regimens were applied at the indicated concentrations for each experiment: Continuous treatment lasted for 72 h. Transient (pulse) treatments lasted for the indicated period of time, followed by five concurrent washes with PBS and addition of TGFβRI inhibitor (EMB Millipore, 616452) to the culture media at a final concentration of 200 nM. For EMT reversion experiments, cells were treated for 72 h, and then TGF-β withdrawn as done as in the transient treatments. For paracrine effect experiments, GFP-labeled NMuMG or EpRAS cells were cultured in 6-well plates (2 × 10^4^ cells/well) and treated with 100 pM TGF-β for 24 h, washed five times with PBS, and overlaid with the unlabeled and non-treated corresponding cell line for 72 h. For mammosphere assays, 5000 cells were plated in ultralow attachment plates (Corning) with the standard sphere media^48^. The number of spheres were counted 6 days later.

### Molecular cloning and CRISPR/Cas9 mediated genome editing

The mouse *Zeb1* gene overexpression construct was generated using the pLEX-MCS-Puro lentiviral vector. cDNA was introduced into pLEX using the SpeI and AgeI cutting sites. PCR primers used were: FW_SpeI_Zeb1 (AGTCACTAGTCATGCAGCGAATGATCCAAC) and Rev_AgeI_ Zeb1 (AGTCACCGGTCACTCTACAATATTCTACTC). The *Cdh1*-EGFP reporter^49^ construct contains the –375/ + 135 *CDH1* promoter region inserted into the pEGFP-N1 plasmid using the AseI and BglII sites. Mouse cDNA was introduced into pLEX using the SpeI and AgeI sites. Deletion of the Z-box-2 Zeb1-binding site (−354/−360) on the *miR-200c/miR-141* promoter was achieved using the CRISPR-Cas9 vector system pLentiCrispr-v2. sgRNA oligo pairs (gRNA 5′-CACCGGGTCAGGCGGGTCTGGTGCC-3′; and reverse complement 5′-AAACGGCACCAGACCCGCCTGACCC-3′) were cloned into the pLentiCrispr-v2. 48 h after transfection with the CRISPR-Cas9 construct, GFP-positive cells are sorted by FACS, seeded as single cells, and expanded into clonal populations. Clones with successful deletion of the Z-box-2 binding site was identified and confirmed by sequencing. All experiments where performed using wild type and mutant clonal populations except for Fig. 1 and Supplementary Fig. 1, in which experiments were done using the parental cell lines.

### Viral production and transduction

Lentiviral plasmids were transfected into HEK293T cells together with the envelope plasmid (VSVG) and gag-pol plasmid (pCMV-dR8.91) following the standard lentiviral packaging protocol to generate lentiviruses. Cells were transduced in culture and selected for the corresponding antibiotic resistance.

### RNA isolation and quantitative real-time PCR

RNA from cell lines was isolated using a mirVana miRNA isolation kit (Ambion) or RNeasy mini kit (Qiagen). mRNA quantification was performed using the Superscript IV First-strand kit (Invitrogen) for cDNA synthesis and the Power SYBR^®^ green PCR master mix (Applied Biosystems) for qPCR. Mature miR-200a, miR-200b, miR-200c, miR-429, and miR-141 were reverse-transcribed using the TaqMan Reverse Transcription Kit (Applied Biosystems) followed by real-time PCR using TaqMan miRNA assays (Applied Biosystems). All analysis was performed using an ABI 7900HT PCR machine according to the manufacturer’s instructions. A standard curve was created from serial dilutions of cDNA for each gene of interest. Values were normalized by the expression of mouse *GAPDH* or *HMBS* for mRNA, or *RNU6B and SnoRNA-142* for miRNA, in each sample. The primers used for mRNA expression analyses are listed in the Supplementary Table 1.

### Western blot analysis

SDS lysis buffer (0.05 mM Tris-HCl, 50 mM BME, 2% SDS, 0.1% Bromophenol blue, 10% glycerol) was used to collect protein from cells. Samples were heat denatured. Protein was equally loaded, separated on a 10% SDS-page gel, transferred onto a pure nitrocellulose membrane (BioRad), and blocked with 5% milk. Primary antibodies for immunoblotting included anti-pSMAD2 (1:1000, Cell Signaling, 3108), anti-pSMAD3 (1:250, ThermoFisher, 44-246 G), anti-SMAD2/3 (1:1000, Cell Signaling, 3102), anti-CDH1 (1:5000, BD Biosciences, 610181), anti-CDH2 (1:2000, BD Biosciences, 610920), anti-ZEB1 (1:3000, Bethyl, A301-922A), anti-Vimentin (1:5000, BD Biosciences, 550513), and anti-β-Actin (1:5000, Abcam, ab8227) for loading control. Membranes were incubated with horseradish peroxidase (HRP)-conjugated anti-mouse secondary antibody (1:5000, GE Healthcare) or anti-rabbit secondary antibody (1:5000, GE Healthcare) for 1 h, after which chemiluminescent signals were detected using ECL substrate (GE Healthcare).

### Immunofluorescence analysis and imaging

Cells were cultured on untreated coverslips in 6-well plates (2 × 10^4^ cells/well) and treated as indicated. Coverslips were fixed for 30 min in methanol at −20 °C, washed once with acetone, and then 5 times with PBS. Samples were blocked and permeabilized for 30 min in blocking buffer (5% normal goat serum, 0.3% TritonX-100 in PBS). Cells were incubated with anti-CDH1 antibody (BD Biosciences, 610181; diluted 1:100 in blocking buffer) for 1 h at RT. Secondary antibodies (BD Biosciences, 1:500) were incubated for 1 h at RT. Coverslips were mounted in DAPI mounting media (Vector Labs). Images were obtained using the Zeiss Axiovert 200 microscope with a 10x objective and the AxioVision software version 4.6.3 SP1.

### Histology of mouse lung tissues

For quantification of metastatic lesions, mouse lungs were isolated, washed briefly in PBS, fixed in Bouin’s fixative (Sigma) overnight at 4 °C, and stored in 70% ethanol before counting lung nodules. For histological analysis, lungs were fixed in 10% formalin overnight at 4 °C, dehydrated, and embedded in paraffin (Tissue Tek Embedding Station). 3μm sections were cut on a Leica RM2255 rotary microtome. For antigen retrieval, deparaffinized slides were immersed in R-buffer A (Electron Microscopy Services) and boiled in a microwave for 20 min. Samples were blocked and permeabilized for 30 min in blocking buffer (5% normal goat serum, 0.3% TritonX-100 in PBS), followed by primary antibody incubation overnight at 4 °C using anti-CDH1 (1:100, BD Biosciences, 610181). Secondary antibody (1:500, BD Biosciences) was incubated for 1 h at RT. All sections were analyzed using a Nikon A1 confocal microscope and Zeiss fluorescence microscope.

### Flow cytometry

Cultured cells were lifted using enzyme-free cell dissociation buffer (Gibco), resuspended in FACS buffer (PBS supplemented with 5% newborn calf serum) and filtered through 100 μm nylon cell strainers. Samples were fixed in 4% formaldehyde for 10 min at 4 °C and permeabilized in ice cold methanol for 10 min; this fixation/permeabilization step is critical for the cell population distribution analysis. This was followed by staining with anti-CDH1-alexa488 antibody (1:200, Cell Signaling, 3199) for 30 min. Finally, cells were analyzed on a FACSort instrument (BD Biosciences).

### Transwell invasion assay

Invasion assays were performed as previously described^17^. Briefly, inserts containing 8 μm pores (Costar) were coated with 50 μL growth-factor-reduced Matrigel (1 mg/mL, Corning) and allowed to solidify overnight at 37 °C. The following day, cells were trypsinized, washed in PBS, and resuspended in serum-free media. 10^5^ cells were seeded on the inserts, which were then placed in wells with serum-containing media. 24 h post-seeding, the cells that had invaded into the wells were trypsinized, pelleted via centrifugation, resuspended in 100 μL media, and counted on a hemocytometer.

### Microarray analysis

Wild type and mutant EpRAS cells were treated with 100 pM of TGF-β. Control cells were treated with the corresponding volume of PBS. After 3 days of continuous treatment, total RNA was prepared from cells using the RNeasy mini kit (Qiagen). The expression of mRNA in mouse cell lines was determined with the Agilent Mouse GEv1 8 × 60 K Microarray (G4852A). The mRNA microarray analyses were performed using a two color system. Briefly, the RNA samples and universal mouse reference RNA (Agilent 740100) were labeled with CTP-cy5 and CTP-cy3, respectively, using the Agilent Quick Amp Labeling Kit. Labeled testing and reference RNA samples were mixed in equal proportions, and hybridized to the arrays as described above. After hybridization, the mRNA arrays were scanned with an Agilent G2565BA scanner and raw data were extracted using Agilent Feature Extraction software (v10.7). Data were analyzed using the GeneSpring GX software (Agilent). The expression value of mRNA array refers to the Log2(Cy5/Cy3) ratio.

### Gene set enrichment analysis (GSEA)

Normalized microarray Log2 ratio expression data generated from EpRAs cells were rank-ordered by differential expression between cell populations using a fold change metric. Multiple probes for the same gene were collapsed into one value by the highest probe reading when there were fewer than 3 probes match, and median when there were 3 or more probes matches. Interrogated signatures from the MSigDB v5.1 release include those relating to Hallmark_EMT (M5930), miR-200s targets (TarBase), Lim_MaSC_Up (M2573), and Naba_ECM_Glycoproteins_UP (M3008). Gene signatures were tested using default enrichment GSEA statistics and compared to enrichment results from 1000 random permutations to obtain p-value, q-value, and normalized enrichment score (NES).

### Statistical analysis

Results are presented as mean ± standard error of the mean (SEM), as indicated in figure legends. BLI signals were analyzed by nonparametric Mann–Whitney test. All other comparisons were analyzed by unpaired, two-sided, independent Student’s *t*-test without equal variance assumption, unless otherwise described in figure legends. Asterisks denote *p*-value significance: *p < 0.05; **p < 0.01; ***p < 0.005. Non-parametric datasets were evaluated using the Mann–Whitney *U* test. For metastasis-free survival analysis, Kaplan–Meier plots and significance with *P* log-rank test were used. All of the experiments with images (BLI, FACS, immunofluorescence, western blot) were repeated >3 times and representative images are shown.

### Code availability

All computer code are scripts in Python or Mathematica. The scripts implement methods are described in the Supplementary mathematical analysis (Supplementary Tables 2-20) and are available from the corresponding authors upon request.

## Electronic supplementary material

Supplementary Information

Reporting Summary

## Data Availability

All microarray data generated in this study have been deposited at the NCBI Gene Expression Omnibus with the accession code GSE106533(https://www.ncbi.nlm.nih.gov/geo/query/acc.cgi?acc=GSE106533). Other gene sets used for GSEA analysis are found in the MSigDB database v5.1 release under the code: M5930, M2573, and M3008. The KM plotter breast cancer dataset^36^ is obtained from http://kmplot.com/analysis. All other data supporting the findings of this study are available from the corresponding authors upon request. A Reporting Summary for this Article is available as a Supplementary Information file.
