# Author Correction: Hysteresis control of epithelial-mesenchymal transition dynamics conveys a distinct program with enhanced metastatic ability

**DOI:** 10.1038/s41467-019-08509-2

**Published:** 2019-01-28

**Authors:** Toni Celià-Terrassa, Caleb Bastian, Daniel D. Liu, Brian Ell, Nicole M. Aiello, Yong Wei, Jose Zamalloa, Andres M. Blanco, Xiang Hang, Dmitriy Kunisky, Wenyang Li, Elizabeth D. Williams, Herschel Rabitz, Yibin Kang

**Affiliations:** 1 Department of Molecular Biology, Princeton University, Princeton, NJ 08544 USA; 2 Program in Applied and Computational Mathematics, Princeton University, Princeton, NJ 08544 USA; 3 Lewis-Sigler Institute of Integrative Genomics, Princeton University, Princeton, NJ 08544 USA; 4 Department of Mathematics, Princeton University, Princeton, NJ 08544 USA; 5 School of Biomedical Sciences, Translational Research Institute, Queensland University of Technology (QUT), Brisbane, QLD 4102 Australia; 6 Department of Chemistry, Princeton University, Princeton, NJ 08544 USA; 7 Present Address: Cancer Program, IMIM (Hospital del Mar Medical Research Institute), 08003 Barcelona, Spain

Correction to: *Nature Communications*; 10.1038/s41467-018-07538-7; published online 27 Nov 2018.

The original version of this Article contained an error in the spelling of the author Daniel D. Liu, which was incorrectly given as Daniel Liu. This has now been corrected in both the PDF and HTML versions of the Article.

