# Supplementary material for: Hysteresis control of epithelial-mesenchymal transition dynamics conveys a distinct program with enhanced metastatic ability
Source: Nat Commun. 2018 Nov 27;9:5005. doi: 10.1038/s41467-018-07538-7 (PMC6258667; doi:10.1038/s41467-018-07538-7)
Supplement: Supplementary file 1 — Supplementary Information [file 41467_2018_7538_MOESM1_ESM.pdf]

Supplementary Figures and Tables

Supplementary Notes

Hysteresis control of epithelial-mesenchymal transition dynamics conveys a distinct program with enhanced metastatic ability

by Toni Celià-Terrassa et al.

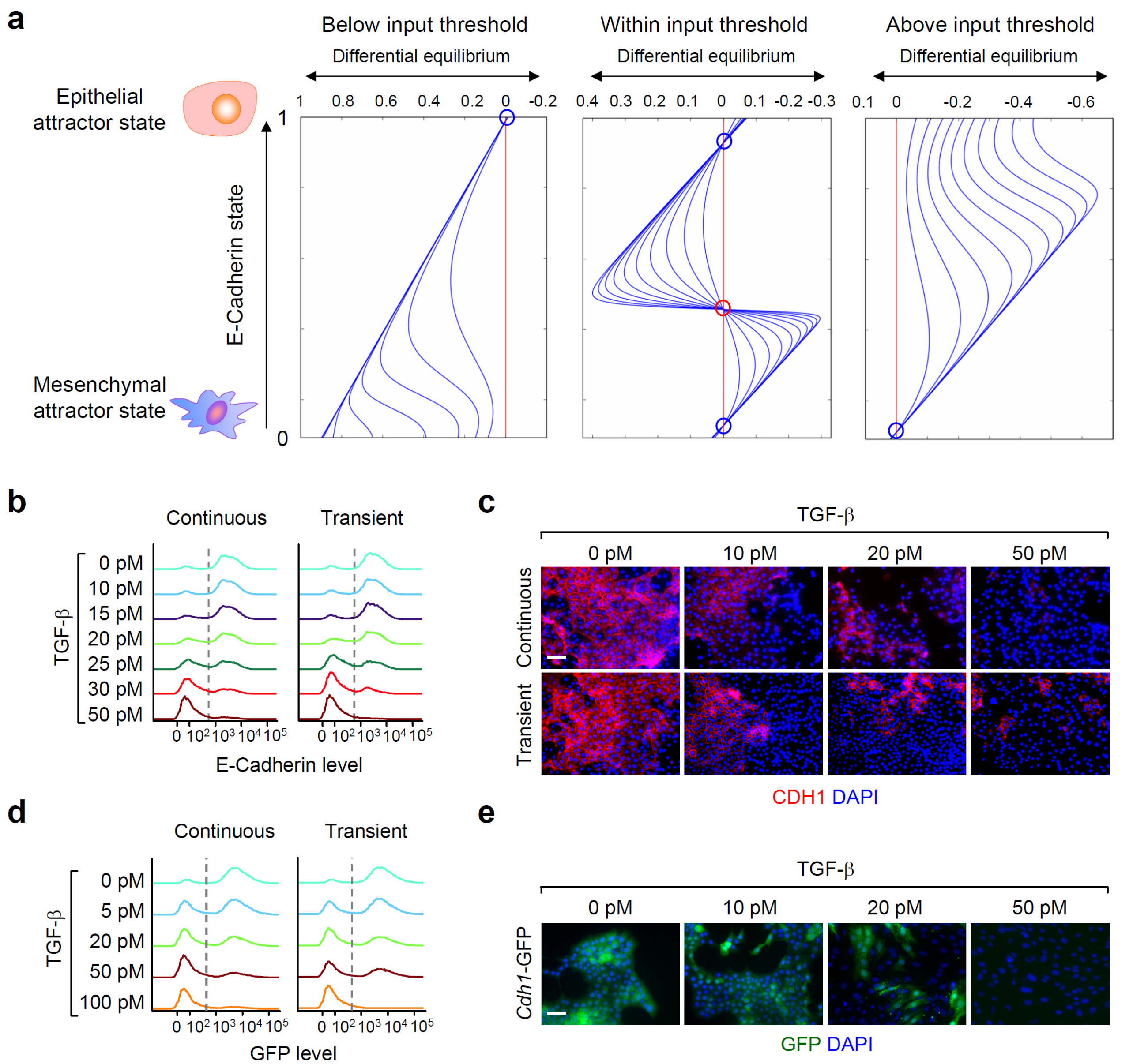

Supplementary Figure 1. Hysteresis attractors and experimental validation. (**a**) Forward orbits of the iterated function system for TGF-β stimulus below, within, and above threshold. The E-Cadherin (phenotype) forward orbits of the iterated function system, conveyed as a sequence of blue curves whose intersections with the red axes occur at stable (blue circles) or unstable (red circle) equilibria. Ten iterations each are shown across three values of TGF-β (T): below threshold (T=1), within threshold (T=4), and above threshold (T=7). The red axes are all situated on [0,1], ranging over mesenchymal and epithelial phenotypes. (**b, c**) Flow cytometry (**b**) and immunofluorescence (**c**) analyses of the endogenous CDH1 expression in EpRAS cells after treatment with the indicated concentrations of TGF-β for 72 hours (histograms, left), or for just 1 hour of transient treatment, followed by measurement of CDH1 expression 72 hours later (histograms, right). (**d, e**) Flow cytometry (**d**) and immunofluorescence (**e**) analyses of the *Cdh1*-promoter driven GFP reporter activity (green) in NMuMG cells after continuous treatment with indicated concentrations of TGF-β for 72 hours (histograms, left), or for just 1 hour of transient treatment, followed by measurement of GFP expression 72 hours later (histograms, right). Scale bars: 40 μm.

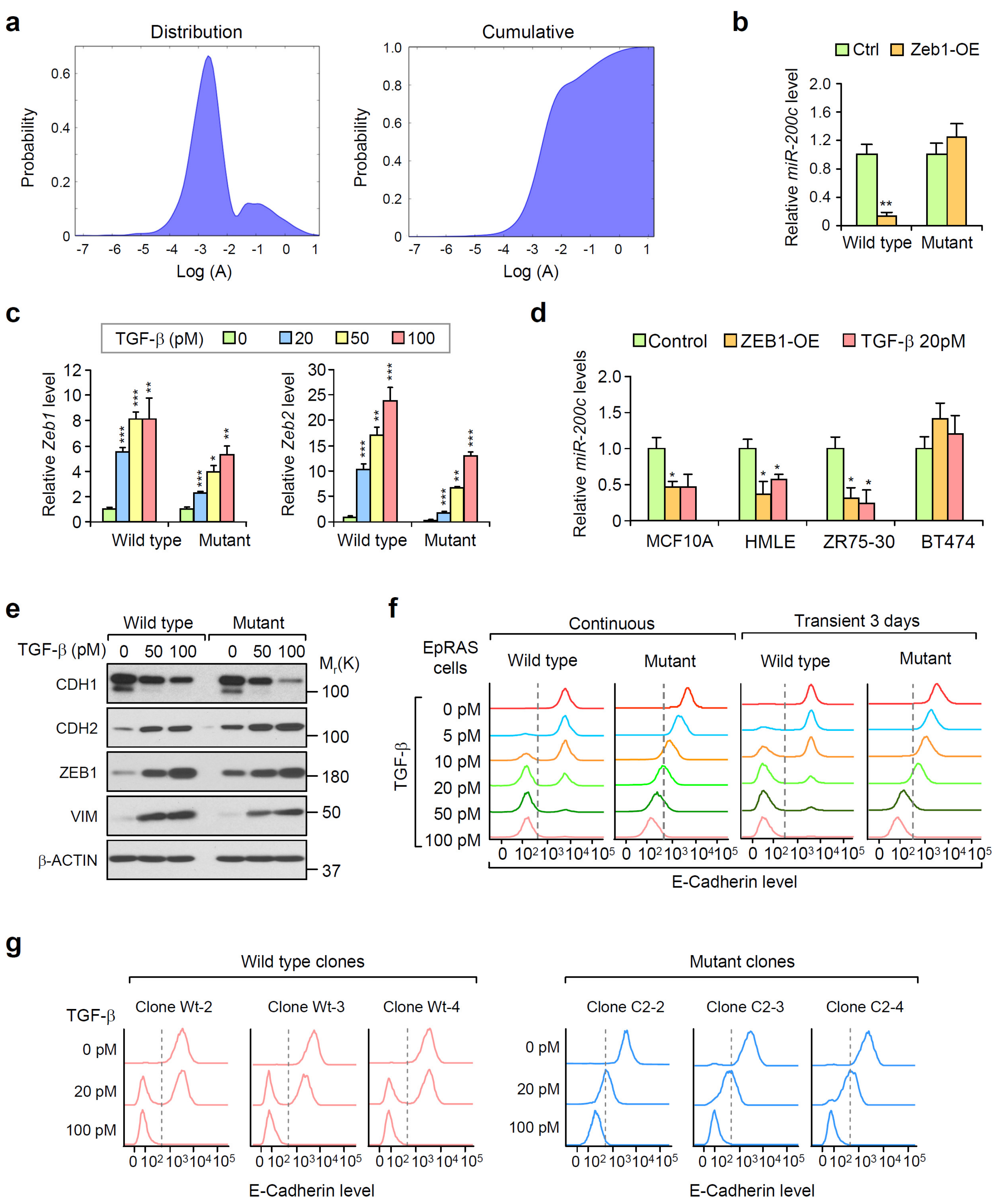

Supplementary Figure 2. Hysteresis identification, validation and regulation of miR-200s expression. (**a**) Probability density and empirical distribution functions of log A, where A is the area of the hysteresis region. These functions are computed using 10,000 (independent) random parameter vectors. (**b**) MiR-200c expression quantified by qRT-PCR analysis with ectopic overexpression of Zeb1 (n=3 biological replicates) in NMuMG cells. (**c**) Expression of

Zeb1 and *Zeb2* in wild type vs. mutant NMuMG cells after 72 hours of continuous treatment of TGF-β at the indicated concentration. Data represent mean ± SEM. n=4 biological replicates. (**d**) *miR-200c* expression in different cell lines after overexpression of Zeb1 or 72 hours of continuous 20 pM TGF-β treatment. Data represent mean ± SEM. n=3 biological replicates. (**e**) Western blot analysis of EMT markers in wild type and mutant EpRAS cells treated at the indicated concentration of TGF-β for 72 hours. (**f-g**) Flow cytometry analysis of CDH1 expression in wild type and mutant EpRAS cells after 72 hours (continuous) or transient (1 hour) TGF-β treatment (**f**), and in multiple clones of wild type and mutant NMuMG cells with or without 72 hours of TGF-β treatment at the indicated concentration (**g**). * $P<0.05$, ** $P<0.01$, *** $P<0.001$ by two-tailed Student's *t*-test in **b**, **c**, **d**.

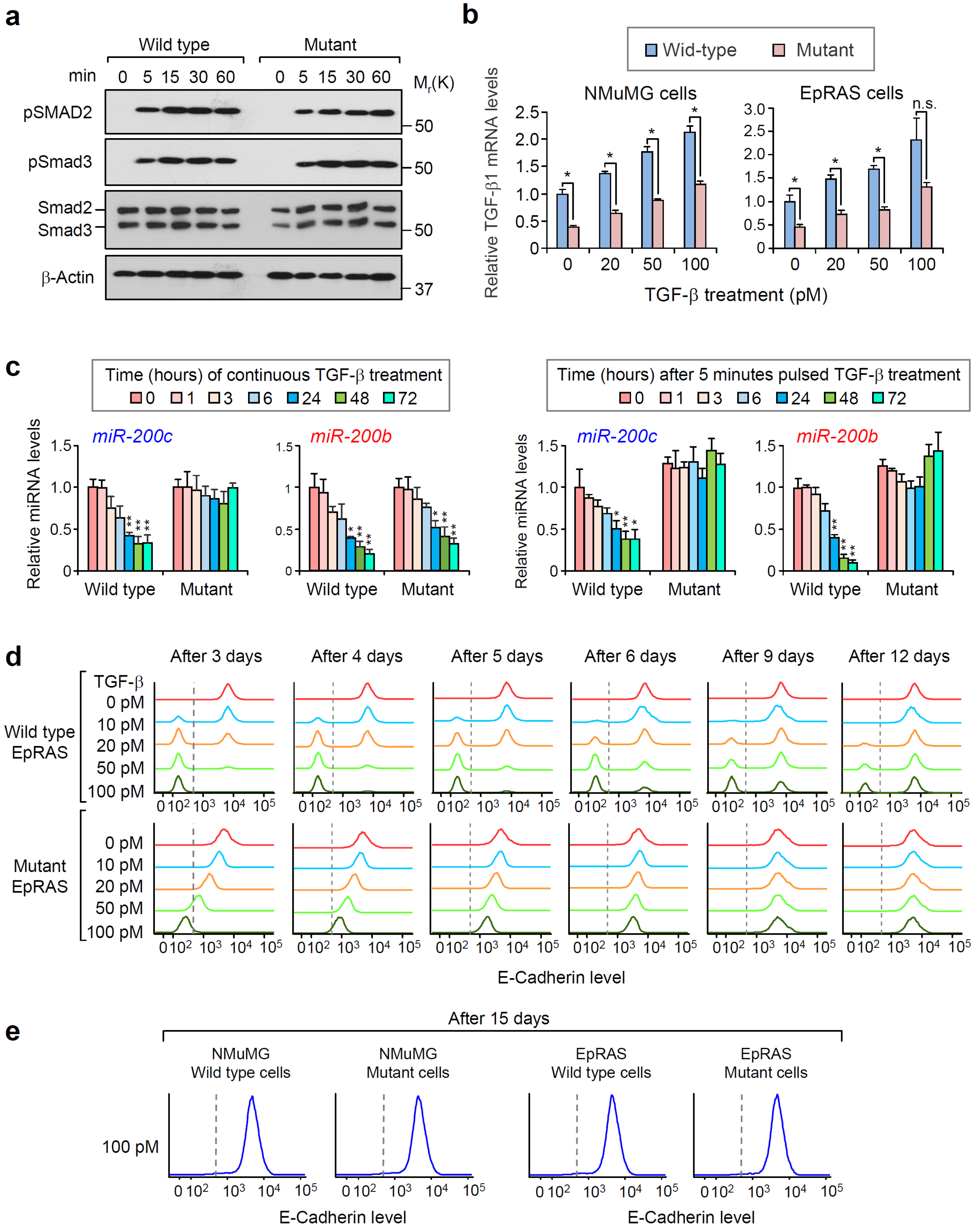
a
Wild type
Mutant
min
0 5 15 30 60
0 5 15 30 60
Mr(K)
pSMAD2
pSmad3
Smad2
Smad3
β-Actin
50
50
50
37
b
Wid-type
Mutant
NMuMG cells
EpRAS cells
Relative TGF-β1 mRNA levels
n.s.
TGF-β treatment (pM)
c
Time (hours) of continuous TGF-β treatment
0 1 3 6 24 48 72
Time (hours) after 5 minutes pulsed TGF-β treatment
0 1 3 6 24 48 72
miR-200c
miR-200b
Relative miRNA levels
Wild type
Mutant
d
After 3 days
After 4 days
After 5 days
After 6 days
After 9 days
After 12 days
TGF-β
0 pM
10 pM
20 pM
50 pM
100 pM
Wild type EpRAS
Mutant EpRAS
E-Cadherin level
e
After 15 days
NMuMG Wild type cells
NMuMG Mutant cells
EpRAS Wild type cells
EpRAS Mutant cells
100 pM
E-Cadherin level
E-Cadherin level

Supplementary Figure 3. EMT dynamics and reversion. (**a**) Western blot analysis of phospho-Smad2, phospho-Smad3, total Smad2 and total Smad3 in NMuMG cells treated with 100 pM TGF-β for the indicated duration. (**b**) *TGF-β1* expression measured by qRT-PCR in wild type and mutant NMuMG and EpRAS cells after 72h of continuous TGF-β treatment at the indicated concentrations. n=3 biological replicates. (**c**) qRT-PCR analysis of *miR-200c* and *miR-200b* expression at the indicated times after continuous or a 5 minute pulse treatment of 100 pM TGF-β. n=3 technical replicates. (**d, e**) Flow cytometry analysis of the CDH1 expression showing the reversion of wild type and mutant EpRAS cells (**d**), NMuMG and EpRAS cells (**e**) after 72 hours of treatment with the indicated concentrations of TGF-β, followed by withdrawal for indicated duration. Data represent mean ± SEM in **b** anc **c**. * *P*<0.05, ** *P*<0.01 by two-tailed Student's *t*-test in **b** and **c**.

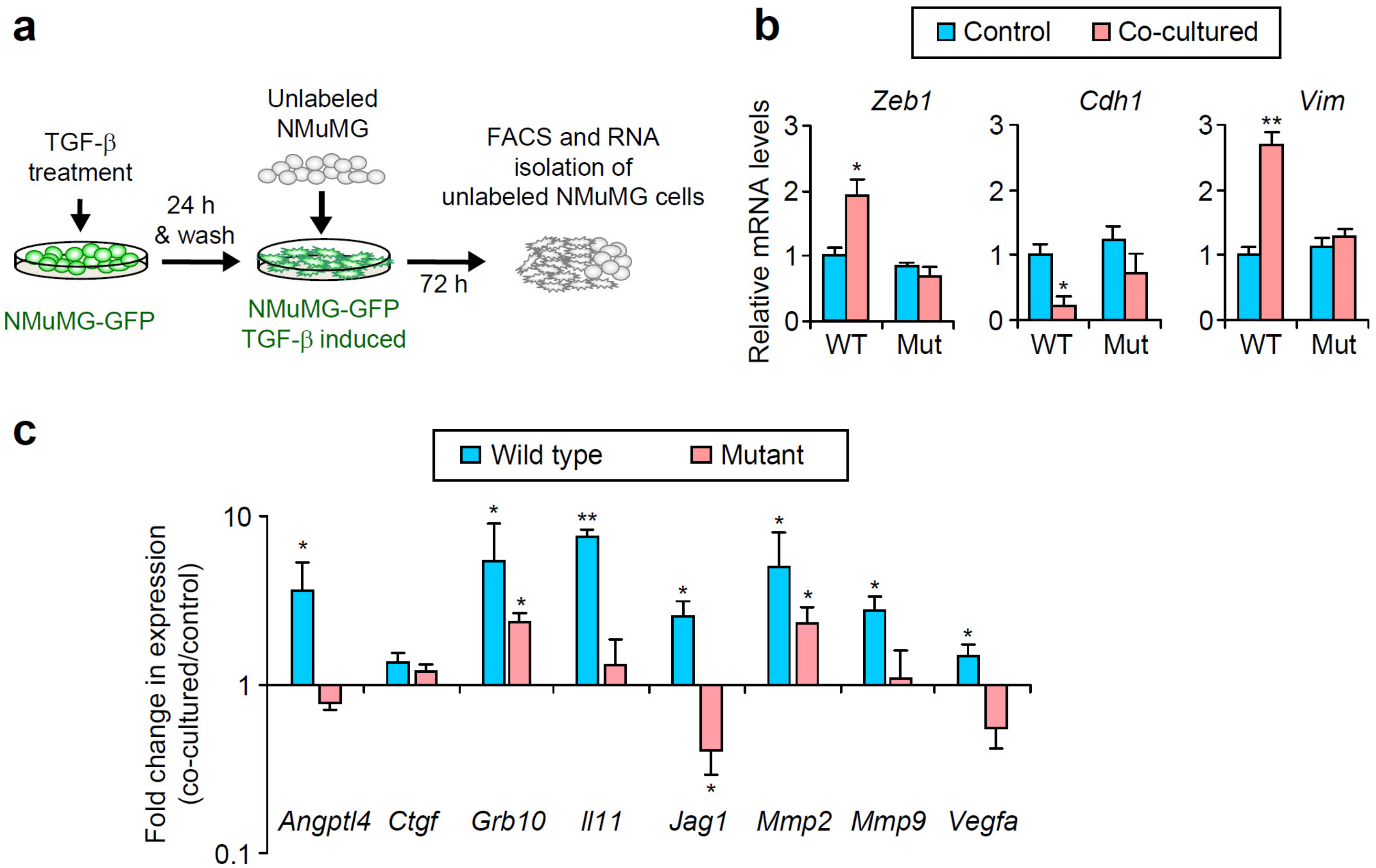

Supplementary Figure 4. Co-cultured effects in wild type and mutant NMuMG cells. (**a**) Schematic representation of co-culture procedure of NMuMG cells. NMuMG-GFP cells were induced with 100 pM TGF-β for 24 hours, after which TGF-β was washed off and unlabeled NMuMG cells were added into the culture. After 72 hours of co-culture, GFP-negative cells were separated by flow cytometry for qRT-PCR analysis. (**b, c**) qRT-PCR analyses of the expression of *Zeb1*, *Cdh1* and *Vim*, as well as TGF-β target metastatic genes (**c**) in wild type and mutant GFP-negative NMuMG cells. Data represent mean ± SEM in **b** and **c**. n=3 biological replicates. * *P*<0.05, ** *P*<0.01 by two-tailed Student's *t*-test in **b** and **c**.

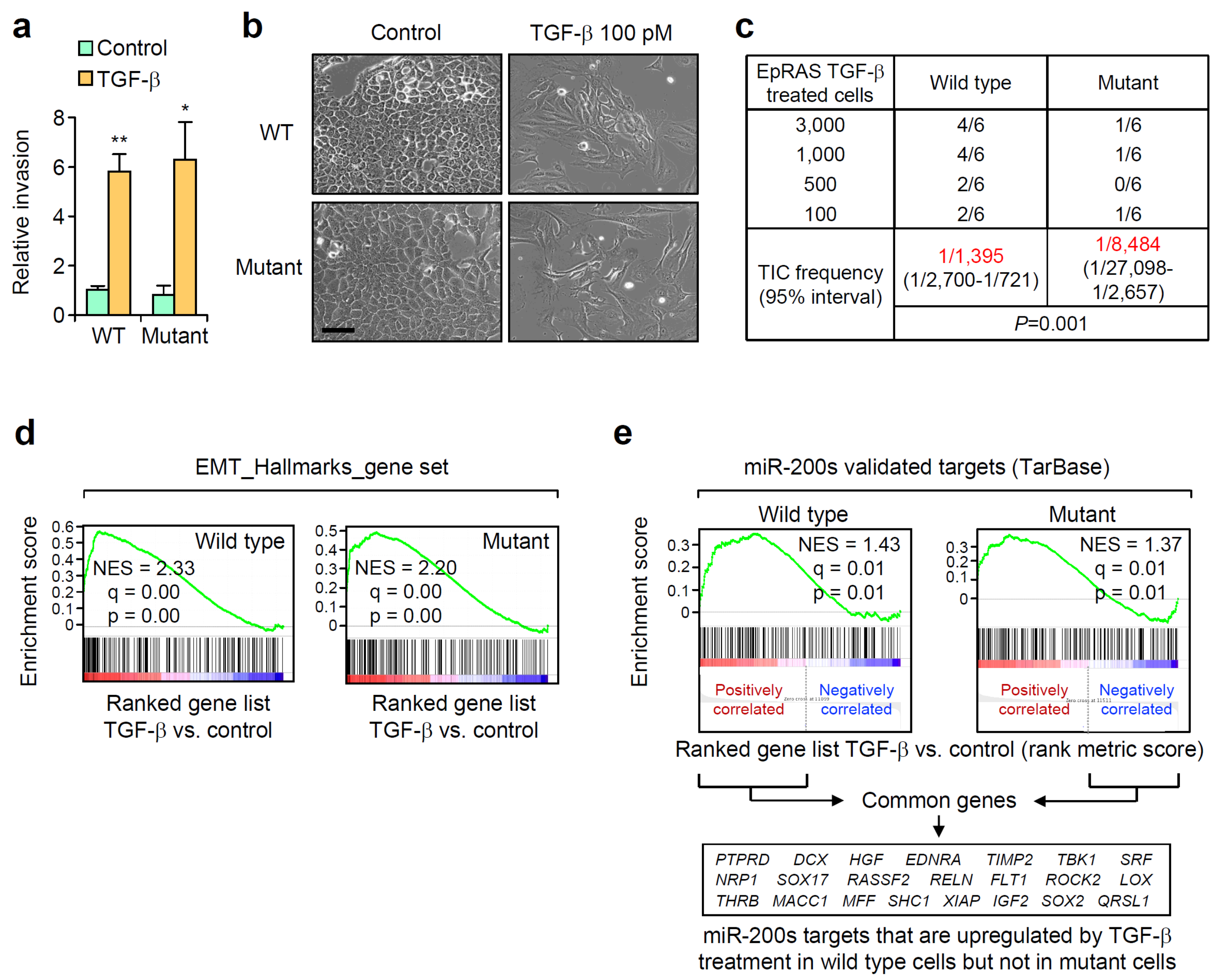

Supplementary Figure 5. Characterization of EMT in wild type and mutant cells. (**a**) Quantification of invaded cells in transwell Matrigel invasion assays 24 hours after seeding of 100,000 NMuMG cells with the indicated conditions of TGF-β. n=3 biological replicates; Data represent mean ± SEM. * P<0.05, ** P<0.01 by two-tailed Student's t-test. (**b**) Phase contrast images of NMuMG cells after 3 days of treatment with 100pM of TGF-β. Scale bar: 100 μm. (**c**) Tumor take rate upon mammary fat pad (MFP) injection of the indicated number of wild type and mutant EpRAS cells after treatment with 100pM of TGF-β for 72 h. n = number of MFP injections as indicated in the table. Tumor initiating cell (TIC) frequency calculated by the ELDA software is indicated in red. (**d-e**) GSEA demonstrating the enrichment of the gene sets from the MSigDB related to EMT (M5930) (**d**) and miR-200s validated targets in TarBase (**e**) in the ranked gene list of TGF-β treated vs. control conditions in wild type or mutant EpRAS cells. The list of genes in (**e**) represent those miR-200 target genes that are upregulated in wild type cells by TGF-β (Fold change > 0 by rank metric score), but not in mutant cells (Fold change < 0 by rank metric score). NES, normalized enrichment score.

Supplementary Table 1. List of primers used in the study.

Gene	Forward	Reverse
mZeb1	GCTGGCAAGACAACGTGAAAG	GCCTCAGGATAAATGACGGC
mZeb2	ATTGCACATCAGACTTTGAGGAA	ATAATGGCCGTGTCGCTTCG
mCdh1	CAGGTCTCCTCATGGCTTTGC	CTTCCGAAAAGAAGGCTGTCC
mSnai1	CACACGCTGCCTTGTGTCT	GGTCAGCAAAAGCACGGTT
mCdh2	TTACAGCTACCTGCCACTTTTC	CCAGCAGATTTCAAGGTGGAC
mVimentin	CGTCCACACGCACCTACAG	GGGGGATGAGGAATAGAGGCT
mFn1	TGTGTGGGGAACGGTCGTGGA	TGGCACTGGTCAATGGGGTCACA
mTGFb1	TGGAGCCTGGACACACAGTA	TGTGTTGGTTGTAGAGGGCA
mAngptl4	CATCCTGGGACGAGATGAACT	TGACAAGCGTTACCACAGGC
mCtgf	GACCCAACTATGATGCGAGCC	CCCATCCCACAGGTCTTAGAAC
mGrb10	GGATTCGCGTTCCGAGACC	ACGGAGAAAAACCGAAGGCAA
mIl11	TGCTGACAAGGCTTCGAGTAG	ACATCAAGAGCTGTAAACGGC
mJag1	CCTCGGGTCAGTTTGAGCTG	CCTTGAGGCACACTTTGAAGTA
mMmp2	ACCTGAACACTTTCTATGGCTG	CTTCCGCATGGTCTCGATG
mMmp9	CTTCCGCATGGTCTCGATG	CAGGCCGAATAGGAGCGTC
mVegfa	AAAGGCTTCAGTGTGGTCTGAGAG	GGTTGGAACCGGCATCTTTATC
mHmbs	CGGGAAAACCCTTGTGATGC	CTCAGAGAGCTGGTTCCCAC
mGapdh	AGGTCGGTGTGAACGGATTTG	TGTAGACCATGTAGTTGAGGTCA

Supplementary Notes

1 Mathematical analysis of TGF-β induced epithelial to mesenchymal transition dynamics

Mathematical models and biological contexts (Figs. 1-4)

Supplementary section(s)	Figure(s)	Mathematical model	Biological context
1.1, 1.2, 1.3, 1.4, 2.1	Fig. 1b, Fig. S1a	Ordinary differential equation (ODE)	Single cell; deterministic transcriptional model
1.5, 2.2	Fig. 1c, d; Fig. 3e	Ensemble of ODE's	Homogeneous collection of cells; deterministic transcriptional model with random parameters and initial conditions
2.4	Fig. 2b, Fig. S2a	High dimensional model representation (HDMR)	Global sensitivity analysis of hysteresis bifurcation of ODE
2.5	Fig. 3f, Fig. 4a, b	ODE and partial differential equation (PDE)	Multi-cellular; deterministic transcriptional model; spatial diffusion; random subset of dosed cells

Code availability

All computer code are scripts in Python and Mathematica. The scripts implement the methods described in this document and are available upon request.

1.1 From biology to a general ODE-based mathematical model

We identify a general ODE-based mathematical model to the biological mechanisms (Fig. 1a). To simplify presentation, we list species and their abbreviations, corresponding to (Figs. 1a, b), below in Supplementary Table 2. The biological mechanisms and corresponding mathematical representations are exhibited in Supplementary Table 3.

Supplementary Table 2: Species and abbreviations identified to (Figs. 1a, b)

Species	Abbreviation
TGFb	T
TGFbR1	R
Smad2	$S2$
Smad3	$S3$
Smad2•P	$S2P$
Smad3•P	$S3P$
Smad4	$S4$
Smads	S
miR-200 mRNA	mM
miR-200	M
ZEB1/2 mRNA	mZ
ZEB1/2	Z
E-cadherin mRNA	mE
E-cadherin	E
miR-200•ZEB1/2 mRNA	MmZ

* The symbol "•" indicates *complexing*.

Supplementary Table 3: Biological mechanisms and general mathematical representations of (Fig. 1b)

Biological Mechanism	Mathematical Representation	Description and Notes
TGFb $\nearrow$ TGFbR1	$\frac{d\text{R}}{dt} = a(T) - b(R)$	TGF-β induced activation of TGFb receptor I
Smad2 $\xrightarrow{\text{TGFbR1}}$ Smad2 • P	$\frac{dS2P}{dt} = c(S2, R) - d(S2P)$	Receptor activation of Smad
Smad3 $\xrightarrow{\text{TGFbR1}}$ Smad3 • P	$\frac{d\text{S3P}}{dt} = c(S3, R) - d(S3P)$	Receptor activation of Smad
Smad2 • P + Smad3 • P + Smad4 $\rightarrow$ Smads	$\frac{dS}{dt} = e(S2P, S3P, S4) - f(S)$	Smad complexing
Smads $\nearrow$ ZEB1/2 mRNA $\nearrow$ ZEB1/2	$\frac{dmZ}{dt} = n(TF_Z, S) - p(MmZ) - q(mZ)$	Smad transcriptional activation of ZEB [1, 2]
miR-200 + ZEB1/2 mRNA $\rightarrow$ miR-200 • ZEB1/2 mRNA	$\frac{dMmZ}{dt} = r(M, mZ) - s(MmZ)$	miR-200 post transcriptional repression of ZEB
ZEB1/2 $\searrow$ miR-200 mRNA $\searrow$ miR-200	$\frac{dmM}{dt} = v(TF_M, Z) - w(mM)$	ZEB transcriptional repression of miR-200
ZEB1/2 $\searrow$ E-cadherin mRNA $\searrow$ E-cadherin	$\frac{dmE}{dt} = x(TF_E, Z) - y(mE)$	ZEB transcriptional repression of E-cadherin

* The symbol "$\nearrow$" means *leads to increase in*.
* The symbol "•" indicates *complexing*.
* The symbol "$\searrow$" means *leads to decrease in*.
* The symbol "$\rightarrow$" means *leads to*.

Taking the general ODE-model of Supplementary Table 3, we simplify by assuming negligible delays between transcription and translation (gene and protein expression respectively), enabling treatment of individual species. Hereafter, model efforts are concerned with gene expression. For the reduced set of equations characterizing gene regulation, we prescribe Michaelis-Menten-style kinetics. This model is based on the following assumptions: (1) transcriptional activation is proportional to the number of binding sites occupied by transcriptional activators and (2) transcriptional repression is proportional to the transcriptional repressors bound. We have the following considerations for the transcriptional output of gene G:

- exogeneously influenced by only transcriptional activation and repression, with respective rates $k_{\mathsf{G},a}, k_{\mathsf{G},r} \in \mathbb{R}^+$ ("reaction rate")
- for activation : $i = 1, 2, ..., n_{\mathsf{G}} < \infty$ different types of binding domains (E-box, Z-box, etc), each binding domain type being bound by $m_{\mathsf{G},i} \in \mathbb{N}$ different transcription factors (TF) and each binding domain type having $\ell_{\mathsf{G},i} \in \mathbb{N}$ binding domains
- for repression : $v = 1, 2, ..., s_{\mathsf{G}} < \infty$ different types of repression domains, each repression domain type bound by $q_{\mathsf{G},v} \in \mathbb{N}$ different repressors and each repression domain type having $r_{\mathsf{G},v} \in \mathbb{N}$ binding domains
- for each domain (activation or repression), each transcriptional factor is identified to some binding constant $K \in \mathbb{R}_+$ ("Michaelis-Menten constant")
- decay rate $d_{\mathsf{G}} \in \mathbb{R}^+$
- baseline transcription rate $B_{\mathsf{G}} \in \mathbb{R}^+$

The model form is then

$$\frac{d\mathsf{G}}{dt} = B_{\mathsf{G}} + \underbrace{k_{a_{\mathsf{G}}} \prod_{i=1}^{n_{\mathsf{G}}} \Big(\sum_{j_i=1}^{m_{\mathsf{G},i}} \Big(\frac{(\mathsf{TF}_{j_i}/\mathsf{K}_{j_i})^{\ell_{\mathsf{G},i}}}{1 + \sum_{j_i=1}^{m_{\mathsf{G},i}} (\mathsf{TF}_{j_i}/\mathsf{K}_{j_i})^{\ell_{\mathsf{G},i}}} \Big) \Big)}_{\text{activation}}$$

$$+ \underbrace{k_{r_{\mathsf{G}}} \prod_{v=1}^{s_{\mathsf{G}}} \Big(\sum_{p_v=1}^{q_{\mathsf{G},v}} \Big(\frac{1}{1 + \sum_{p_v=1}^{q_{\mathsf{G},v}} (\mathsf{TF}_{p_v}/\mathsf{K}_{p_v})^{r_{\mathsf{G},v}}} \Big) \Big)}_{\text{repression}} - d_{\mathsf{G}}\mathsf{G}$$

$$\leq B_{\mathsf{G}} + k^*_{a_{\mathsf{G}}} + k^*_{r_{\mathsf{G}}} - d_{\mathsf{G}} \sup \mathsf{G} < \infty$$

where $k^*_{a_{\mathsf{G}}} = k_{a_{\mathsf{G}}} \prod_{v=1}^{n_{\mathsf{G}}} m_{\mathsf{G},i}$ and $k^*_{\mathsf{G},r} = k_{\mathsf{G},r} \prod_{v=1}^{s_{\mathsf{G}}} q_{\mathsf{G},v}$.

1.2 Application of ODE-based mathematical model to TGF-β induced EMT signaling

We denote Smads, ZEB1/2, E-cadherin, and miR-200 as S, Z, E, and M respectively, and note that the putative promoter for miR-200 has two E-box binding sites and two Z-box binding sites, identified to two clusters. Both Smad•X, where X is a co-repressor ("•" means *complexing*, Smad•X means *Smad recruitment of corepressor X*), and ZEB1/2 bind to E-box sites [3, 4, 5, 6], and Z-box binding sites are exclusively bound by ZEB1/2 [7]. Therefore, miR-200 repression is competitive for Smad•X and ZEB1/2 for E-box binding, and this E-box binding is synergistic with Z-box binding. We introduce a simple quadratic term to account for the complexing reaction between miR-200 and ZEB1/2, a key component of the mutual inhibitory feedback loop [5].

Definition 1 (EMT model). *We denote the EMT system as $\dot{x} = f(x, \theta)$ with state variables $x \in \mathbb{R}^4_+$, parameters $\theta \in \mathbb{R}^{17}_{>0}$, and initial conditions $x_0 \in \mathbb{R}^4_{\geq 0}$. The system is specified as*

$$\frac{dS}{dt} = B_S + \frac{k_S T}{K_T + T} - d_S S$$
$$\frac{dZ}{dt} = B_Z + \frac{k_Z S}{K_S + S} - k_{MZ} M Z - d_Z Z$$
$$\frac{dM}{dt} = B_M + k_M \left(\frac{K_Z}{K_Z + Z}\right)\left(\frac{1}{1 + \left(\frac{Z}{K_Z}\right) + \left(\frac{S}{K_S}\right)}\right) - k_{MZ} M Z - d_M M$$
$$\frac{dE}{dt} = B_E + \frac{k_E K_Z}{K_Z + Z} - d_E E,$$

with species

S : *Smad*
Z : *ZEB1/2*
M : *microRNA-200 (miR-200)*
E : *E-cadherin (CDH-1),*

where for species "X" we have B_X as baseline transcription rates, k_X as reaction coefficients, and K_X as Michaelis-Menten constants. k_{MZ} denotes the reaction rate for the complexing reaction between miR-200 and ZEB1/2.

1.3 Mathematical results for the TGFb EMT model of Definition 1

Recall that the symbol " □" indicates the end of a proof. In this section we use tools from dynamical systems theory, in particular fixed-point analysis of non-linear ODE models. For more information on these ideas, see [8]. For concision, the contents of the mathematical proofs of this section are detailed in (later) Supplementary Section 3.

We show that there exists a solution to the mathematical model for TGFb EMT.

Theorem 1 (Global existence of a solution to model of Definition 1). *Consider the system $\dot{x} = f(x, \theta)$ from Definition 1 with $B_X = 0$. Then, there exists an attractive fixed-point for every θ and x_0.*

Proof. Supplementary Section 3. □

There are several important properties of the fixed points of the system salient in the proof. Firstly, the parameters K_T, k_S, d_S, and T only affect the fixed point value of S, and S is completely determined by their values. So, when we are only interested in fixed point properties, it is possible to take S to be a system parameter rather than T, which lowers the dimension of the parameter space by three. The range of possible values of S for fixed parameters and varying T is $[0, \frac{k_S}{d_S}]$, so we define $S_{\max} = \frac{k_S}{d_S}$. Similarly, the fixed point values of Z and M are bounded by $\frac{k_Z}{d_Z}$ and $\frac{k_M}{d_M}$ respectively, so let us call these $Z_{\max}$ and $M_{\max}$ respectively. Furthermore, the sequences $\{(F \circ G)^n\}_{n=1}^{\infty}$ and $\{(G \circ F)^n\}_{n=1}^{\infty}$ are not uniformly convergent. This does not affect the zeros themselves, but for certain θ and x_0, the zeros are isolated points. This behavior can be observed in Fig. S1a for the intra-threshold panel.

In discussing stability we will make use of the Jacobian of f, given by

$$J_f(x) = \begin{pmatrix} -d_S & 0 & 0 & 0 \\ \frac{k_Z K_S}{(K_S+S)^2} & -k_{MZ}M - d_Z & 0 & -k_{MZ}Z \\ 0 & -\frac{k_E K_Z}{(K_Z+Z)^2} & -d_E & 0 \\ -\frac{k_{MZ}}{K_S}\left(\frac{K_Z}{K_Z+Z}\right)\left(\frac{1}{(1+\frac{Z}{K_Z}+\frac{S}{K_S})^2}\right) & -\frac{k_M}{(K_Z+Z)(1+\frac{S}{K_S}+\frac{Z}{K_Z})}\left(\frac{K_Z}{K_Z+Z}+\frac{1}{1+\frac{S}{K_S}+\frac{Z}{K_Z}}\right) - k_{MZ}M & 0 & -k_{MZ}Z - d_M \end{pmatrix}.$$

It is immediately evident that $-d_S$ and $-d_E$ are both negative eigenvalues of $J_f(x)$. The sum of the other two eigenvalues is still negative since both $-k_{MZ}M - d_Z$ and $-k_{MZ}Z - d_M$ are negative, so there is at least one more eigenvalue with negative real part. Thus, $J_f(x)$ has at least three eigenvalues with negative real part. Consequently, we can use the determinant of $J_f(x)$ to determine asymptotic stability.

Lemma 1 (Asymptotic stability of model of Definition 1)**.** *Denoting $f(x) = f(x, \theta)$, a fixed point $f(x_0) = 0$ is asymptotically stable if and only if $\det J_f(x_0) > 0$.*

Proof. Supplementary Section 3. □

The following result is useful because it shows that the stabilities of the equilibria coincide for the differential system and the iterated function system.

Theorem 2 (Correspondence)**.** *An equilibria of the system $\dot{x} = f(x)$ is asymptotically stable if and only if the corresponding fixed-point of $F \circ G$ is asymptotically stable.*

Proof. Supplementary Section 3. □

We should note that Theorem 2 does not state that the stable equilibria are identical for the differential and iterated function systems for a given θ and x_0. Although this is generally the case, we observe that for some values of θ and x_0 the corresponding stable equilibria take dramatically different values.
The determinant of $J_f(x)$ can be simplified by expanding by minors across the row $-d_S$ and then down the column of $-d_E$, giving

$$\det(J_f) = d_S d_E \begin{vmatrix} \frac{\partial f_Z}{\partial Z} & \frac{\partial f_Z}{\partial M} \\ \frac{\partial f_M}{\partial Z} & \frac{\partial f_M}{\partial M} \end{vmatrix}.$$

Putting

$$A_f = \begin{pmatrix} \frac{\partial f_Z}{\partial Z} & \frac{\partial f_Z}{\partial M} \\ \frac{\partial f_M}{\partial Z} & \frac{\partial f_M}{\partial M} \end{pmatrix}$$

as before and noting $d_S, d_E > 0$, we observe that $\det(J_f)$ has the same sign as $\det(A_f)$.

Corollary 1. *An equilibrium of the system $\dot{x} = f(x)$ is stable if and only if A_f evaluated at the point has positive determinant.*

Lemma 2 (Stability)**.** *$\dot{x} = f(x)$ is asymptotically stable at the fixed-point $x = (S, Z, M, E) \in \mathbb{R}_+ \times \{0\} \times \mathbb{R}_+ \times \mathbb{R}_+$ for any positive θ and is asymptotically stable at the fixed-point $x = (S, Z, M, E) \in \mathbb{R}_+ \times (0, \infty) \times \mathbb{R}_+ \times \mathbb{R}_+$ for any positive $\theta \setminus k_M$ and $0 < k_M < C(\theta^*, S, Z, M)$, where C is a constant depending on $\theta^* = (k_{MZ}, k_M, K_S, K_Z, d_M, d_Z)$.*

Proof. Supplementary Section 3. □

Corollary 2 (Instability)**.** *$\dot{x} = f(x)$ is unstable at the fixed-point $x \in X$ if and only if $Z > 0$ and $k_M > C(\theta^*, S, Z, M)$.*

An important finding is the nature of the input-output relationship for the differential system. Here, we show that the output-input relationship is unique; however, the converse is not true. To show this, we first derive a polynomial relation that gives the equilibrium values of Z.

Proposition 1 (Polynomial relation for Z)**.** *The equilibrium values of Z are given by the roots of a quartic polynomial. Expressed as a polynomial relation, this is*

$$\frac{k_{MZ}}{k_Z} Z = \left(\frac{k_{MZ}}{k_M} Z + \frac{1}{M_{max}}\right)\left(1 + \frac{Z}{K_Z}\right)\left(1 + \frac{Z}{K_Z} + \frac{S}{K_S}\right)\left(\frac{S}{K_S + S} - \frac{Z}{Z_{max}}\right).$$

Proof. Supplementary Section 3. □

We consider only the roots whose corresponding values satisfy $(E, M, Z) \in (0, E_{\text{max}}) \times (0, M_{\text{max}}) \times (0, Z_{\text{max}})$, so-called *biologically feasible* values of (E, M, Z).

Proposition 2. *No single biologically feasible value of Z is an equilibrium value of Z for two distinct positive values of S.*

Proof. Supplementary Section 3. □

This result means that there is a one-to-one mapping from some subset of values of Z to S. Said another way, if there exists one value of $S > 0$ that produces $Z \in (0, Z_{\text{max}})$, then there exists no more. The result reveals that the mapping from Z to S is either one-to-one or one-to-none.

Theorem 3 (Z to S)**.** *For every $Z \in (0, Z_{max})$, there exists a unique positive S.*

Proof. Supplementary Section 3. □

There are one to one relationships between Z and E and S and T, specified through $E = E_{\text{max}} \frac{K_Z}{K_Z + Z}$ and $S = S_{\text{max}} \frac{T}{K_Z + T}$, solved to give $T = \frac{K_T}{\frac{S_{\text{max}}}{S} - 1}$.

Corollary 3 (E to T)**.** *For every $E \in (0, E_{max})$, there exists a unique positive T.*

The converse of Theorem 3 (and Corollary 3) is not true, as multistability, where a single equilibrium of S maps to multiple equilibria of Z (and M), occurs for certain parameters $\theta_h \subset \theta \subseteq \mathbb{R}^{13}_{>0}$. Enforcing $Z \in (0, Z_{\text{max}})$ has the effect of making some of the roots of Z inaccessible. Namely, the smallest root is negative, $Z < 0$, and a branch of equilibria $Z > Z_{\text{max}}$ occurs. These are all impermissible as they mathematically occur outside $(0, Z_{\text{max}})$. In terms of $T \mapsto E$, for θ_h, as we vary T as observe the following cases

1. when $T \leq a$, then $E_1 = E_2 < 0$ and $0 \leq E_3 = E_4 \leq E_0$
2. when $T \in (a, b)$, then $E_1 < 0$ and $0 < E_2 < E_3 < E_4 < E_{\text{max}}$
3. when $T \geq b$, then $E_1 < 0$ and $0 < E_2 < E_{\text{max}}$ and $E_4 \geq E_3 > E_{\text{max}}$.

The number of distinct realizable values of $E \in [0, E_{\text{max}}]$ is one or three, and the particular $T \mapsto E$ correspondence is determined by the initial conditions. In the one-to-three mapping consisting of $0 < E_2 < E_3 < E_4 < E_{\text{max}}$ where $T \in (a, b)$, E_2 and E_4 are stable fixed-points and E_3 is unstable. Unfortunately, despite the simple appearance of the differential system, necessary and sufficient conditions for this bifurcation do not appear to be analytically expressible, and we proceed with numerical treatment of the composition and properties of θ_h.

Definition 2 (Hysteresis bifurcation)**.** *Consider the system $\dot{x} = f(x, \theta_h)$. The bifurcation of E_∞ in T is called a hysteresis bifurcation.*

1.4 Simplification of mathematical model of Definition 1

Because the model exhibits a one-to-one correspondence between the steady-state value of S and the parameter T, we simplify the EMT model.

Definition 3 (Simplified EMT model)**.** *We simplify the model from Definition 1 by replacing the state variable S with the parameter T,*

$$\frac{dZ}{dt} = \frac{k_Z T}{K_T + T} - k_{MZ} M Z - d_Z Z$$

$$\frac{dM}{dt} = k_M \left(\frac{K_Z}{K_Z + Z}\right) \left(\frac{1}{1 + \left(\frac{Z}{K_Z}\right) + \left(\frac{T}{K_T}\right)}\right) - k_{MZ} M Z - d_M M$$

$$\frac{dE}{dt} = \frac{k_E K_Z}{K_Z + Z} - d_E E.$$

We denote state variables as

$$x = (Z, M, E) \in [0, 1]^3,$$

parameters as

$$\theta = (T, k_Z, k_M, k_E, k_{MZ}, K_T, K_Z, d_Z, d_M, d_E) \in \mathbb{R}^{10}_{>0},$$

and initial conditions as $x_0 = (Z_0, M_0, E_0) \in \{0, 1\}^3$.

We use the model from Definition 3 to define the relation $g : T \mapsto E_\infty$ between the dose of TGFb protein, T, and the steady-state mRNA concentration of CDH-1, E_∞.

1.5 Extensions to the mathematical model of Definition 3

Random parameters

Consider the system $\dot{x} = f(x, \theta)$ with $(x_0, \theta) \in X \times \Theta$. In real biological systems, uncertainty exists in (x_0, θ), where cell-to-cell variation occurs in the counts of molecules and proteins, cell size, receptor affinities (from counts and spatiotemporal fluctuations of bound receptors on cell surface), and so on. Hence, (x_0, θ) is defined as a random variable on the probability space $(\Omega, \mathscr{H}, \mathbb{P})$.

Autocrine regulation

We introduce additional state variables (T_f, T_b) into the ODE model from Definition 3 and replace the occurrence of T by T_b to provision a simple model for autocrine regulation.

Definition 4 (Autocrine model)**.** *Let T_f denote free (unbound) TGFb and T_b denote TGFb that is bound to a receptor. Dynamics are specified through the system*

$$\frac{dT_f}{dt} = \frac{k_a T_b}{K_a + T_b} - \frac{k_b T_f}{K_b + T_f} - d_{T_f} T_f$$

$$\frac{dT_b}{dt} = \frac{k_b T_f}{K_b + T_f} - d_{T_b} T_b.$$

Free TGFb (T_f) has the following fates: production by secretion, loss by conversion to T_b, and loss by biological decay. Bound TGFb (T_b) has the following fates: production by conversion from T_f and loss by biological decay.

Partial differential equation model for spatial diffusion

We conceptualize a single cell located at A on some domain $D \subset \mathbb{R}^2$ acting as a point source of T_f, with auto-induced secretion of T_f on A by the activities of T_b. We define a partial differential equation model for spatial diffusion.

Definition 5 (EMT model with diffusion)**.** *Consider models from Definitions 3 and 4, and endow the state variable T_f with diffusion on the 2D spatial domain $D \subset \mathbb{R}^2$ (note that the model from Definition 1 can be similarly equipped with diffusion, retaining S as a state variable). For a single cell associated with domain $A \subset D$, we specify the simplified EMT model having diffusion as*

$$\begin{aligned}
\frac{dT_f}{dt} &= c\Delta T_f + \delta_A \left(\frac{k_a T_b}{K_a + T_b} - \frac{k_b T_f}{K_b + T_f} \right) - d_{T_f} T_f \\
\frac{dT_b}{dt} &= \frac{k_b T_f}{K_b + T_f} - d_{T_b} T_b \\
\frac{dZ}{dt} &= \frac{k_Z T_b}{K_T + T_b} - k_{ZM} ZM - d_Z Z \\
\frac{dM}{dt} &= k_M \left(\frac{K_Z}{K_Z + Z} \right) \left(\frac{1}{1 + \left(\frac{Z}{K_Z}\right) + \left(\frac{T_b}{K_{T_b}}\right)} \right) - k_{ZM} ZM - d_M M \\
\frac{dE}{dt} &= \frac{k_E K_Z}{K_Z + Z} - d_E E
\end{aligned}$$

We note that the variables (T_b, Z, M, E) take non-zero values only on (are restricted to) A. For simplicity, we denote $X = X(t, r) = T_f$, $Y = Y(t) = T_b$ with a 1-D Laplacian $\Delta = \frac{\partial}{\partial r^2}$ and take $A = \{0\}$, giving

$$\begin{aligned}
\frac{dX}{dt} &= c\Delta X + \delta_A \left(\frac{k_a Y}{K_a + Y} - \frac{k_b X}{K_b + X} \right) - d_X X \\
\frac{dY}{dt} &= \frac{k_b X(t, A)}{K_b + X(t, A)} - d_Y Y
\end{aligned}$$

for initial conditions $X(0, r) = d \cdot \delta_A$ and $Y(0) = 0$ and with $X \in C^{1,2}(\mathbb{R}_+ \times \mathbb{R}_+)$ such that $X : \mathbb{R}_+ \times \mathbb{R}_+ \to \mathbb{R}_+$ is governed by the above PDE, while $Y \in C^1(\mathbb{R}_+)$ such that $Y : \mathbb{R}_+ \to \mathbb{R}_+$ is governed by an ODE on $A = \{0\}$. *This is a 1-D heat equation where the boundary term is a source that fluctuates based on the value of the PDE at the boundary.* For $r = 0$, we have

$$\frac{dX_t(0)}{dt} = c\Delta X_t(0) + \frac{k_{auto} Y_t}{K_{auto} + Y_t} - \frac{k_{bind} X_t(0)}{K_{bind} + X_t(0)} - d_{T_f} X_t(0)$$

The above system can be solved using the *method of lines* using the idea of discretizing the spatial domain, which generates a system of ODEs that can be numerically integrated using the standard techniques [9]. We approximate the diffusion term using a second-order central finite difference scheme (from Taylor's formula) for interior points

$$\Delta X_t \approx \frac{X_t^{(i+1)} - 2X_t^{(i)} + X_t^{(i-1)}}{(\Delta r)^2} + O((\Delta r)^2)$$

where $O((\Delta r)^2$ is the truncation error and $i = 2, ..., M$, and for the boundary point $i = 1$, a slightly different second-order finite difference scheme

$$\Delta X_t \approx \frac{X_t^{(i+1)} - 2X_t^{(i)} + X_t^{(i+1)}}{(\Delta r)^2} + O((\Delta r)^2).$$

In this research, we use a two-dimensional second-order finite difference scheme. This model is exhibited in Fig. 4a with annotations of biological dynamics.

2 Simulated dynamics and computational studies

The models in Supplementary Section 1 are particular instances of ordinary and partial differential equation models of transcription, with random parameters and initial conditions. Despite their simple functional form, they exhibit non-trivial dynamics, including bifurcations representing hysteresis having complex functional dependence on model parameters. To understand how some measure of the hysteresis bifurcation depends on the model parameters, we compute a *high dimensional model representation* (HDMR), which reveals the hierarchy of functional effects (referred to as *component functions*). Using the HDMR component functions, we perform a *global sensitivity analysis* (GSA). Importantly, both the HDMR expansion and its associated GSA may be computed from a *single* set of random input-output data.

2.1 Identification of the hysteresis bifurcation in E-cadherin expression

We take

$$(d_Z, d_M, d_E) \leftarrow (k_Z, k_M, k_E),$$

imposing an upper bound of one across the corresponding state variables, with initial conditions $(Z_0, M_0) \leftarrow (0, 1)$. Using parameter values

$$(k_Z, k_M, k_E, k_{MZ}, K_T, K_{Z'}, K_{Z''}) \leftarrow (10^{-1}, 1, 1, 100, 1, 10^{-1}, 10^{-1}),$$

corresponding to a robust miR-200-ZEB1/2 regulatory axis, fixed-point iteration is conducted with $\epsilon = 10^{-4}$. The forward and backward orbits of $g : T \mapsto E_\infty$ are exhibited in Fig. 1b ranging over $T \in [0, 10]$. The blue curve is generated with $E_0 \leftarrow 1$, the green curve with $E_0 \leftarrow E_\infty(T)$, and region between is the region of hysteresis, containing the equilibria of unstable fixed-points (“separatrix” or “boundary”).

Definition 6 (Bistability)**.** *The existence of two disconnected concentration “levels” for CDH1 as TGFb varies in Fig. 1b is called bistability.*

Definition 7 (Hysteresis)**.** *The difference between the locations of the discontinuities of the forward and backward orbits as TGFb varies in Fig. 1b for CDH1 is called hysteresis.*

The asymptotic stability analysis from Supplemental Section 1.2 reveals two regimes of behavior: two eigenvalues of the Jacobian are always negative, and the third eigenvalue’s sign depends on θ and T. The separatrix of these regimes in θ and T defines a hysteresis bifurcation: as we vary the parameters T and θ and initial conditions X_0 for the map $X \mapsto f(X, T, \theta) - X$, we move from a single attractive fixed-point to two attractive fixed-points and an intermediary repelling fixed-point. This phenomenon is illustrated with the behavior of the iterated function system

$$\{(F \circ G)^n(Z \mapsto E) - (Z \mapsto E) : n = 1, \ldots, 10\}, \quad T \in \{1, 4, 7\}$$

in Fig. S1a.

2.2 Identification of bistability in E-cadherin expression

Recall the probability space $(\Omega, \mathscr{H}, \mathbb{P})$. We let the parameter vector

$$\theta \equiv (k_Z, k_M, k_E, k_{MZ}, K_T, K_{Z'}, K_{Z''}) \in \Theta$$

be a random variable with distribution (image measure) ν. We specify ν as a product of gamma measures with parameters (α, β), where $\mathbb{E}\,\theta = \alpha\beta$ and $\mathbb{V}\text{ar}\,\theta = \alpha\beta^2$, and N iid random realizations are attained

$$X = \{\theta_i \sim \nu(\Theta) : \text{for all } i \in \mathbb{N}_N\}\,.$$

To illustrate "hysteresis" the (hyperparamter) values of α and β are set such that

$$\mathbb{E}\,\theta = (10^{-1}, 1, 100, 1, 10^{-1}, 10^{-1})$$
$$\mathbb{V}\text{ar}\,\theta = (\frac{\mathbb{E}\,\theta}{10})^2$$

over an equispaced grid $T \in [0, T_{\text{max}}]$ with

$$T_{\text{max}} = 20.$$

Recall the fixed-point mapping, $T \times \Theta \mapsto \mathbb{R}_+$, which takes some value of TGFb , parameter vector θ, and convergence threshold ε, and returns a vector in $X = [0,1]^3$ representing the equilibrium (the fixed-point). Now, consider the trace of the mapping onto $E \subset X$. The notation $E(T, \theta)$ means the value in E depends on the value of TGFb T and the random variable θ. Fig. 1c exhibits the density of $E(T, \theta)$ with superimposed $\mathbb{E}\,E(T, \cdot)$ for $T \in [0, T_{\text{max}}]$, revealing a bimodal distribution. Bimodality is exhibited in switching behavior, whereby the system transitions from epithelial to mesenchymal state, or from mesenchymal to epithelial (MET) state (hence Fig. 3e is a mirror image to Fig. 1c). Fig. 1d illustrates the density of $E(T, \theta)$ for various T profiles. Having held T constant for any value of E, we examine the scenario of a step function input, $T = T(t) = a1_{[t_a, t_b]}(t) + b$, where $a, b > 0$ are positive constants. Numerically integrating the ODE system, we show the distributions of the simulated equilibria in Fig. 1d. Notice that the solutions attained for the ODE system and the corresponding iterated function system are similar for constant and transient inputs. For "non-hysteresis" we set $K_{Z'} = 1$ and keep the remaining parameters at their values. Their results are also shown in Fig. 1d. Bootstrapped population mean estimates with one standard deviation envelopes are exhibited in Fig. 1e.

2.3 Defining a (functional) measure of hysteresis

We define a (non-negative) *measure* of hysteresis as

$$A(\theta) \equiv \int (E_b(t, \theta) - E_a(t, \theta))\,\mathrm{d}t,$$

where, for the TGFb value t, the quantity $E_b(t, \theta)$ is the fixed-point value of E with $X_0 = (0, 1, 1)$ and $E_a(t, \theta)$ is the fixed-point value of E with $X_0 = (1, 0, 1)$. The quantity $A(\theta)$ is the difference of the two solution curves of $E(\theta)$ (identified to the two initial conditions) and computes the area of the interior region formed by these curves ("hysteresis region"). This quantity is illustrated in Fig. 1b, which additionally exhibits the separatrix of unstable equilibria (green dotted; "boundary"). Here, we are interested in examining the structure of the input-output map $\theta \mapsto A(\theta)$, where $\theta = (k_Z, k_M, k_E, k_{MZ}, K_T, K_{Z'}, K_{Z''}) \in \Theta \subseteq \mathbb{R}^7_{>0}$, with $K_Z = (K_{Z'}, K_{Z''})$ for repression of M

and E by Z respectively (without loss of generality, this refinement has cost of one additional parameter). In determination of steady-state, we consider the sequence $(E_n)_n$ to have converged when the difference of successive iterations $E_{n+1} - E_n$ has a L^2 norm less than ε. We set the parameter space to be a scaled uniformly distributed hypercube,

$$\Theta = [0,1] \times [0,100] \times [0,2] \times [0,2] \times [0,3] \times [0,1] \times [0,1].$$

It is important to note that ODE system is uniformly bounded for all time under equality constraints of the parameters, and the bound is specifiable. We enforce unit bounds (in expectation), so that values of $A(\theta)$ are on the same scale and directly comparable. 10,000 independent random realizations of θ were attained using Uniform(Θ). For each random realization, the quantity $A(\theta)$ is estimated using trapezoidal integration with $T \in [0, 20]$ over a equispaced grid of size 200 with $\varepsilon = 10^{-6}$. This collection is denoted by $\mathbf{D} = \{(x_i, y_i)\}$. Fig. S2a reveals the distribution of A (in log-scale) to be bimodally distributed with only a small fraction of Θ admitting any significant amount of hysteresis. Because A takes values over many scales, the log-modulus transform is applied in examining the structure of A in θ,

$$h : \theta \mapsto \log |1 + A(\theta)|.$$

2.4 High dimensional model representation (HDMR)

The quantity $h(\theta)$ is a complex, strongly non-linear surface in θ. Even with the simple model of Definition 1, necessary and sufficient conditions for the existence of hysteresis as analytic relations among the parameters do not appear feasible or straightforward to attain. This is a common setting in dynamical systems, where non-linearity confers great complexity and subtlety to dynamics. This is also a common problem for identification of complex systems from empirical data. Therefore, we think of $h(\theta)$ as a complex system, and we interrogate it by evaluating it on random values in the parameter space, forming the collection

$$\mathbf{D} = \{(x_i = \theta_i, y_i = h(\theta_i))\}.$$

Using $\mathbf{D}$, we profile the functional dependence of h in θ using high dimensional model representation, an analysis tool from the *complexity sciences*, which may be regarded as a kind of uncertainty quantification methodology. Because the method is critical to attaining insight into the behavior of the quantity $h(\theta)$, we give a reasonably detailed overview of the method before prior to application. In doing so, we use the notations of [10] and [11].

Description of HDMR

High dimensional model representation (HDMR) is a finite and non-orthogonal representation of multivariate functions, as a hierarchy of projections into subspaces of increasing dimensions, with the expectation that subspace contributions to output(s) rapidly diminish with increasing dimension. We concentrate here on the essential issues of HDMR as a finite decomposition of the space of square-integrable functions,

$$F = L^2(X, \mathscr{X}, \nu),$$

where $(X, \mathscr{X}, \nu)$ is a (non-degenerate) probability space, with $n = |X| \in \mathbb{N}$ and $p = |F| \in \mathbb{N}$. The inner product $\langle \cdot, \cdot \rangle$ on F, induced by ν, is defined as

$$\langle g, h \rangle \equiv \int_X g(x)h(x)\,\mathrm{d}\nu(x), \quad g(x), h(x) \in F,$$

and the norm $\|\cdot\|_F$ on F, induced by $\langle\cdot,\cdot\rangle$, is defined as

$$\|f(x)\|_F \equiv (\langle f(x), f(x)\rangle)^{1/2} = \left(\int_X f^2(x)\,\mathrm{d}\nu(x)\right)^{1/2}.$$

Definition 8 (High dimensional model representation [10, 12]). *For every $T \in \mathbb{N}_n$ and $f(x) \in F$, and defining*

$$\mathcal{V}_{i_1\ldots i_l} \equiv \Bigg\{ f \in F : f = f_{i_1\ldots i_l}(x_{i_1},\ldots,x_{i_l}) \text{ is a } l\text{-variate function of the inputs } x_{i_1},\ldots,x_{i_l}$$
$$\text{with } \left(\int_{X_j} f_{i_1\ldots i_l}(x_{i_1},\ldots,x_{i_l})\nu_{i_1\ldots i_l}(x_{i_1},\ldots,x_{i_l})\mathrm{d}x_j : j = i_1,\ldots,i_l\right) = \mathbf{0}\Bigg\},$$

the variational problem

$$\min_u \|f(x) - u\|_F, \quad u \in \mathcal{V}_0 \oplus \sum_i \mathcal{V}_i \oplus \sum_{i_1<i_2} \mathcal{V}_{i_1 i_2} \oplus \ldots \oplus \sum_{i_1<\cdots<i_T} \mathcal{V}_{i_1\ldots i_T}$$

is uniquely minimized with the T-order high dimensional model representation (HDMR)

$$u = f^T(x) = \left(\mathscr{P}_0 + \sum_i \mathscr{P}_i + \sum_{i_1<i_2} \mathscr{P}_{i_1 i_2} + \cdots + \sum_{i_1<\cdots<i_T} \mathscr{P}_{i_1\ldots i_T}\right) f(x),$$

where $\{\mathscr{P}_u\}$ is the collection of (hierarchically-orthogonal) projection operators. We denote

$$f_{i_1\ldots i_l}(x_{i_1},\ldots,x_{i_l}) \equiv \mathscr{P}_{i_1\ldots i_l} f(x) \in \mathcal{V}_{i_1\ldots i_l}$$

as the component function of $f(x)$ in variables $(x_{i_1},\ldots,x_{i_l})$ belonging to the component function subspace $\mathcal{V}_{i_1\ldots i_l} \subset F$.

When $T = n$, the projection operators form a resolution of the unit operator, $\sum_u \mathscr{P}_u = \mathbf{1}$. The notion of *hierarchical-orthogonality* is essential, as this guarantees the existence and uniqueness of the decomposition for general (non-degenerate) ν. Hierarchical-orthogonality is a generalization of mutual orthogonality and requires functions to be orthogonal to only those functions defined on its nested subspaces. For example, for $\nu_{123} \neq \nu_1\nu_2\nu_3$, hierarchical-orthogonality implies that $\langle f_{12}, f_1\rangle = \langle f_{12}, f_2\rangle = 0$ but neither implies $\langle f_1, f_2\rangle = 0$ nor $\langle f_{12}, f_3\rangle = 0$.

Define the residual as

$$\varepsilon_T(x) \equiv f(x) - u = f(x) - f_0 - \sum_i f_i(x_i) - \sum_{i_1<i_2} f_{i_1 i_2}(x_{i_1}, x_{i_2}) - \cdots - \sum_{i_1<\cdots<i_T} f_{i_1\cdots i_l}(x_{i_1},\ldots,x_{i_T})$$

and denote the risk functional

$$\mathcal{J} \equiv \|f(x) - u\|_F = \int_X \varepsilon_T^2(x)\mathrm{d}\nu(x).$$

When $T = n$, we have $\mathcal{J} = 0$ (equality):

$$f(x_1,\ldots,x_n) = f_0 + \sum_i f_i(x_i) + \sum_{i_1<i_2} f_{i_1 i_2}(x_{i_1}, x_{i_2}) + \ldots + f_{1\ldots n}(x_1,\ldots,x_n),$$

equivalently written as

$$F = \mathcal{V}_0 \oplus \sum_i \mathcal{V}_i \oplus \sum_{i_1<i_2} \mathcal{V}_{i_1 i_2} \oplus \ldots \oplus \mathcal{V}_{1\ldots n}.$$

A key property of HDMR is that models are often well approximated, or even exactly represented, using HDMR expansions truncated at low-order ($T \ll n$),

$$f(x) \backsimeq f^T(x) = f_0 + \sum_i f_i(x_i) + \sum_{i_1<i_2} f_{i_1 i_2}(x_{i_1}, x_{i_2}) + \ldots + \sum_{i_1<\cdots<i_T} f_{i_1\ldots i_T}(x_{i_1}, \ldots, x_{i_T}),$$

i.e. *f^T is a reduced-order representation of f*. When the input variables are independent ($\nu = \prod_i \nu_i$), the component functions are *mutually orthogonal* and can be recursively constructed,

$$\begin{aligned} f_{i_1\ldots i_l}(x_{i_1}, \ldots, x_{i_l}) \equiv & \mathbf{M}^{i_1\ldots i_l} f(x) - \sum_{j_1<\cdots<j_{l-1}\subset\{i_1,\ldots,i_l\}} f_{j_1\ldots j_{l-1}}(x_{j_1}, \ldots, x_{j_{l-1}}) \\ & - \sum_{j_1<\cdots<j_{l-2}\subset\{i_1,\ldots,i_l\}} f_{j_1\ldots j_{l-2}}(x_{j_1}, \ldots, x_{j_{l-2}}) - \cdots \\ & - \sum_{j\subset\{i_1,\ldots,i_l\}} f_j(x_j) - f_0 \end{aligned}$$

where $\mathbf{M}^{i_1\ldots i_l} f(x) \equiv \int_{X_{-i_1\ldots i_l}} f(x) \prod_{j\notin\{i_1,\ldots,i_l\}} \mathrm{d}\nu_j(x_j)$. For independent inputs, the HDMR component functions convey a decomposition of model variance,

$$\mathbb{V}\text{ar}\, f = \sum_i \mathbb{V}\text{ar}\, f_i + \sum_{i_1<i_2} \mathbb{V}\text{ar}\, f_{i_1 i_2} + \cdots + \mathbb{V}\text{ar}\, f_{1\ldots n},$$

where

$$\mathbb{V}\text{ar}\, f_u \equiv \int_{X_u} f_u^2(x_u)\mathrm{d}\nu_u(x_u).$$

Normalizing by $\mathbb{V}\text{ar}\, f$, we retrieve *sensitivity indices*

$$1 = \sum_i \mathbb{S}_i + \sum_{i_1<i_2} \mathbb{S}_{i_1 i_2} + \cdots + \mathbb{S}_{1\ldots n},$$

reflecting the relation

$$\mathbb{S}_u \equiv \frac{\mathbb{V}\text{ar}\, f_u}{\mathbb{V}\text{ar}\, f}.$$

This illustrates that the HDMR component functions $\{f_u\}$ convey a decomposition of variance. When normalized, this is known as *global sensitivity analysis*. In this context of independent inputs, the sensitivity indices $\{\mathbb{S}_u\}$ are known as *Sobol sensitivity indices* [13]. In global sensitivity analysis applications, only $\{\mathbb{S}_u\}$ are sought (the $\{f_u\}$ are not required), so the $\{\mathbb{S}_u\}$ are (directly) estimated using specialized sampling [14, 15, 16].

Remark 1 (Kolmogorov's superposition theorem). *It would seem that the best finite representation of multivariate functions should involve only univariate functions and for continuous functions this is true: Kolmogorov's superposition theorem [17] is an existence result that establishes that every multivariate continuous function on the unit cube ($X = [0, 1]^n$) can be represented using a finite number of univariate continuous functions,*

$$f(x) = \sum_{i=1}^{2n+1} \phi_i \circ \left(\sum_{j=1}^{n} \psi_{ij} \circ x_j \right),$$

where ϕ_i are ψ_{ij} are the continuous functions. These functions are also highly non-smooth, greatly restricting their utility for applied settings. Compared to Kolmogorov's result, HDMR supposes a hierarchy of projections into subspaces of increasing *dimensions, and expresses $f(x)$ as a superposition of 2^n functions. While many additional HDMR component functions appear when compared to Kolmogorov's result, most of the HDMR component functions are identically zero, or insignificant, for F of practical interest, i.e., a low effective-dimension, $f(x) \simeq f^T(x)$ for $T \ll n.$*

Illustrative example of a HDMR analysis

Consider the "product function" (or monomial)

$$f(x) = \prod_{i=1}^{n} x_i, \quad \text{iid}\, x, \quad \rho \equiv \sigma/\mu \neq 0.$$

Its variance is given by

$$\mathbb{V}\text{ar}\, f = \mu^{2n} \left(\left(1+\rho^2\right)^n - 1 \right).$$

Its component functions are

$$\begin{aligned} f_0 &= \mathscr{P}_0 f(x) = \mu^n \\ f_i(x_i) &= \mathscr{P}_i f(x) = \mu^{n-1} x_i - f_0 \\ f_{ij}(x_i, x_j) &= \mathscr{P}_{ij} f(x) = \mu^{n-2} x_i x_j - f_i(x_i) - f_j(x_j) - f_0 \\ &\vdots \end{aligned}$$

Consider the k-dimension component function $f_u(x_u)$ ($|u| = k$). It sensitivity index is given by $\mathbb{S}_u = \frac{\rho^{2|u|}}{(\rho^2+1)^n - 1}$, and the k-order sum (the sum of all k dimensional sensitivity indices) is given by

$$p\{k\} \equiv \sum_{i_1 < \cdots < i_k} \mathbb{S}_{i_1 \cdots i_k} = \frac{\binom{n}{k} \rho^{2k}}{(\rho^2+1)^n - 1}$$

(and of course $\sum_k p\{k\} = 1$). Because n is fixed, we concentrate on the numerator, which consists of the (polynomial) number of dimension k subspaces $\binom{n}{k}$ and the (exponential) ρ^{2k}. *Observe that when $\rho < 1$ the explained variance of low dimensional approximations increases*, so that mass of the probability vector $\boldsymbol{p} = (p\{k\} : k = 1, \ldots, n)$ concentrates about small k (exponential suppression beats polynomial growth). As a point of reference, the uniform distribution (a common distribution for a "null hypothesis") on the unit interval has $\rho(\text{Unif}[0,1]) = \frac{\sqrt{3}}{3} \approx 0.58$. This HDMR example generalizes: similar results are observed for sums of elementary symmetric polynomials in n variables,

$$E_n(x) = 1 + \sum_{\substack{k \in \mathbb{N}_n \\ i_1 < \cdots < i_k}} x_{i_1} \cdots x_{i_k},$$

where $\tau \equiv \frac{\sigma}{1+\mu}$ regulates expansion efficiency (instead of $\rho = \sigma/\mu$). This worked example has a suggestive conclusion: HDMR analysis reveals that the effective dimension of the product function (monomial) is regulated by the coefficient of variation of the input distribution.

HDMR has been independently formulated for and applied to seemingly distinct domains

HDMR is discussed as early as [18] in ANOVA analysis and has enjoyed extensive application to statistics. When F is the collection of symmetric functionals of *iid* variables HDMR is known as the *Hoeffding decomposition*, a fundamental object in U-statistics [19]. The univariate terms of HDMR are sometimes known as *Hajek projections* and are extensively used to establish asymptotic normality of various statistics [20]. [10] discusses HDMR as a general decomposition of F where the input space resides in $\mathbb{R}^n$ (*function HDMR*) or in the n-fold product space of arbitrary linear topological function spaces (*functional HDMR*). [10] also discusses F as the collection of functions taking finite value at some point $x \in X$, where ν is the Dirac measure sitting at x (*cut HDMR* or *anchored ANOVA*). These ideas have been further developed to F for general (non-degenerate) measures [12], wherein HDMR is known as *generalized functional ANOVA*, which in turn provisions global sensitivity analysis in terms of *structural and correlative sensitivity indices* [21]. In application to goodness-of-fit settings, e.g., Pearson Chi-square, HDMR identifies and eliminates *information leakage* in "big data" settings [22]. Note that sometimes HDMR is known as the *Hoeffding-Sobol decomposition* [23] or *Sobol decomposition* [24]. See [25] for references on HDMR's earlier history.

HDMR usage for this article

In this article, we use random sampling high dimensional model representation (RS-HDMR) [10, 26, 27]. We truncate the (function) HDMR of h to second-order ($T = 2$) and estimate the sensitivity indices, $\{\mathbb{S}_u : |u| \leq T\}$ from N independent random vectors in Θ with independent inputs ($\nu = \prod_i \nu_i$). The HDMR component functions and sensitivity indices were estimated using 500 bootstrap estimates of the component functions, having bootstrap samples of size 1000 from $\mathbf{D}$ and estimators as systems of (orthogonal) bases [26]. The estimator's coefficient of variation $\rho = \mu/\sigma$ was employed to filter out low-quality estimates (we use greater than two).

HDMR results for this article

Supplemental Table 4 reveals significant first and second order sensitivity indices, whereby well over half of (hysteresis) model variance is constituted by first and second-order effects. The parameter having by far the highest total sensitivity index ($\mathbb{S}_u^{\text{total}} \equiv \mathbb{S}_u + \sum_{v \supset u} \mathbb{S}_v$) is $K_{Z'}$, which corresponds to the ZEB repression of miR-200 expression. Examining the significant component functions whereby $K_{Z'}$ participates, Fig. 2b exhibits the first-order component function in $K_{Z'}$, which has significant positive hysteresis effect when taking small values. Furthermore, containing Fig. 2b exhibits the second-order component function in $K_{Z'}$ and k_{MZ} to have a cooperative hysteresis effects, where large positive contributions occur with potent ZEB repressive action on miR-200 and a rapid rate of miR-200-ZEB complexing.

Supplementary Table 4: Bootstrap estimates of first and second order sensitivity indices

Parameter(s)	$\mathbb{S}$
k_Z	0.1020
$K_{Z'}$	0.2283
$\sum \boldsymbol{X_i}$	0.3303
$(k_Z, K_{Z'})$	0.2445
$(k_{MZ}, K_{Z'})$	0.0499
$\sum \boldsymbol{X_{jk}}$	0.2944
$\sum \boldsymbol{X_i} + \sum \boldsymbol{X_{jk}}$	0.6247

* Values of $\mathbb{S}$ are computed per [26], as described in **Method usage for this article** and satisfy $\rho > 2$

2.5 Hysteresis propagation

Both Fig. 3f and Fig. 4b have similar computational treatment: both solve the system of equations of Definition 5 (based on the ODE systems of Definitions 1 and 3). Using the *method of lines*, Definition 5's model is solved on a 2D square grid (n^2 points)—depicted in Fig. 4a—using second-order finite difference approximations and periodic boundary conditions. The rate of convergence of the system to an equilibria, and in turn chosen to be the "end time" of the simulation, depends upon the parameter values. For both figures, we choose "reasonable" sets of parameter values which exhibit interesting non-equilibrium behavior with computationally short integration times t.

For Fig. 3f, a grid-size of $n = 21$ is taken, and the method of lines generates a system of $2\,205$ ODEs. The parameter values for hysteresis and non-hysteresis panels are shown below in Supplementary Table 5. Denoting the subset of the parameter space Θ which exhibits a hysteresis bifurcation as Θ_h, i.e. $\Theta_h \subset \Theta$, we see that nearly all parameters have no such bifurcation: across all values of such parameters, there exists no subset of values in Θ_h. However, for certain parameters, their parameter spaces may be partitioned into Θ_h and Θ_h^{c}. Initial conditions to this system are specified as: the center grid point takes value $X_0(\cdot) = 10$, with the remaining $X_0(\cdot) = 0$ and all $Y_0 = 0$. Using these initial conditions in conjunction with those of Definition 3, the system of ODEs is numerical integrated on $[0, t]$, with time $t = 100$ chosen because the system is at equilibrium. Non-equilibrium dynamics are contrasted between hysteresis and no hysteresis for various time points.

For Fig. 4b, a grid-size of $n = 51$ is taken, and the method of lines generates a system of $15\,606$ ODEs. The parameter values for hysteresis and non-hysteresis panels are shown below in Supplementary Table 6. Initial conditions to this system are specified as: $N = 70$ grid points are randomly assigned (without replacement) probability law $X_0(\cdot) =$ Gamma$(5, 2 \times 10^{-3})$, with the remaining $X_0(\cdot) = 0$ and all $Y_0 = 0$. Using these initial conditions in conjunction with those of Definition 1, the system of ODEs is numerical integrated on $[0, t]$, with time $t = 100$ chosen because the system is at equilibrium. Non-equilibrium dynamics are contrasted between hysteresis and no hysteresis for various time points.

Supplementary Table 5: Parameter values for Fig. 3f

Parameter(s)	Hysteresis	No Hysteresis
n	21	–
$[0, t]$	$[0, 100]$	–
c	10^{-4}	–
$\triangle$	10^{-2}	–
k_a	5×10^{-1}	–
K_a	1	–
k_b	5×10^{-1}	–
K_b	1	–
d_{T_f}	10^{-1}	–
d_{T_b}	1	–
T_{max}	1	–
Z_{max}	1	–
M_{max}	1	–
E_{max}	1	–
K_T	10^{-2}	–
k_Z	10^{-1}	1
K_Z	10^{-4}	10^{-2}
k_M	1	–
k_{MZ}	100	10
k_E	1	–

* The parameters (k_Z, K_Z, k_{MZ}) admit clearly identifiable partitions of (Θ_i) into Θ_h (hysteresis) and Θ_h^{c} (non-hysteresis) subspaces, while the remaining parameters do not and effectively behave as free parameters which do not alter qualitative behaviors. The parameters $(T_{\text{max}}, Z_{\text{max}}, M_{\text{max}}, E_{\text{max}}, K_a, K_b, d_{T_b}, k_M, k_E)$ are set to "unit" values, whilst the parameters identified to TGFb , conveying spatial effects of hysteresis, are set such that their putative effects concentrate well on the chosen (finite) 2D spatial domain.

Supplementary Table 6: Parameter values for Fig. 4b

Parameter(s)	Hysteresis	No Hysteresis
n	51	–
N	70	–
$[0, t]$	$[0, 100]$	–
c	10^{-3}	–
$\triangle$	10^{-2}	–
k_a	10^{-1}	–
K_a	10^{-1}	–
k_b	1	–
K_b	10^{-1}	–
d_{T_f}	1	–
d_{T_b}	1	–
S_{max}	1	–
Z_{max}	1	–
M_{max}	1	–
E_{max}	1	–
K_T	10^{-2}	–
k_S	1	–
K_S	10^{-1}	–
k_Z	1	–
K_Z	5×10^{-5}	5×10^{-3}
k_M	1	–
k_{MZ}	10^4	10^2
k_E	1	–

* The considerations of these parameters are identical to those of Supplementary Table 5 except that k_Z is unchanged.

3 Proofs for Supplementary Section 1.3

Theorem 1.

Proof. We want to demonstrate the existence of a solution to $f(x, \theta) = 0$ for any θ (and x_0), which is the system of equations

$$
\begin{aligned}
0 &= \frac{k_S T}{K_T + T} - d_S S, \\
0 &= \frac{k_Z S}{K_S + S} - k_{MZ} M Z - d_Z Z, \\
0 &= k_M \left(\frac{K_Z}{K_Z + Z} \right) \left(\frac{1}{1 + \frac{Z}{K_Z} + \frac{S}{K_S}} \right) - k_{MZ} M Z - d_M M, \\
0 &= \frac{k_E K_Z}{K_Z + Z} - d_E E
\end{aligned}
$$

For S and E this can be solved immediately, giving

$$S = \frac{k_S}{d_S} \cdot \frac{T}{K_T + T}, \quad E = \frac{k_E}{d_E} \cdot \frac{K_Z}{K_Z + Z},$$

whereby the fixed point value for S is uniquely specified by the system parameters, and the value for E depends only on Z. It remains to consider Z and M. For Z, we have the expression

$$Z = \frac{k_Z}{d_Z + k_{MZ} M} \cdot \frac{S}{K_S + S}$$

where we call the right-hand side $F(M)$, a function determined by the system parameters. Similarly, for M we obtain

$$M = \frac{k_M}{d_M + k_{MZ} Z} \cdot \frac{K_Z}{K_Z + Z} \cdot \frac{1}{1 + \frac{Z}{K_Z} + \frac{S}{K_S}}$$

where we call the right-hand side $G(Z)$, again a function determined by the system parameters. Using these functions, fixed point values of Z and M must satisfy $Z = F(G(Z)) = (F \circ G)(Z)$. Putting $Z_{\text{max}} = \frac{k_Z}{d_Z}$ and $M_{\text{max}} = \frac{k_M}{d_M}$, we have $Z \in [0, Z_{\text{max}}]$ and $M \in [0, M_{\text{max}}]$. We observe that

- $G(Z)$ and $F(M)$ are analytic, non-negative, strictly decreasing, and satisfy $G(Z) \in [0, Z_{\text{max}}]$ and $F(M) \in [0, M_{\text{max}}]$
- $(F \circ G)(Z)$ and $(G \circ F)(M)$ are analytic, non-negative, strictly increasing, and satisfy $(F \circ G)(Z) \in [0, Z_{\text{max}}]$ and $(G \circ F)(M) \in [0, M_{\text{max}}]$.

Defining

- $(F \circ G)^n \equiv (F \circ G) \circ (F \circ G) \circ \ldots \circ (F \circ G)$
- $(G \circ F)^n \equiv (G \circ F) \circ (G \circ F) \circ \ldots \circ (G \circ F)$

we see that for each $n \in \mathbb{N}$, $(F \circ G)^n$ and $(G \circ F)^n$ are analytic, non-negative, strictly increasing, and satisfy $(F \circ G)^n(Z) \in [0, Z_{\text{max}}]$ and $(G \circ F)^n(M) \in [0, M_{\text{max}}]$. By the Contraction Theorem for analytic functions, we have

- $\forall Z \in [0, Z_{\text{max}}], \lim_{n \to \infty} (F \circ G)^n(Z) = \alpha \in [0, Z_{\text{max}}]$

- $\forall M \in [0, M_{\text{max}}]$, $\lim_{n\to\infty} (G \circ F)^n(M) = \beta \in [0, M_{\text{max}}]$

where α and β are attractive fixed-points of $F \circ G$ and $G \circ F$ in $[0, Z_{max}]$ and $[0, M_{max}]$ for every $Z \in [0, Z_{\text{max}}]$ and $M \in [0, M_{\text{max}}]$. □

Lemma 1.

Proof. Note that x_0 is asymptotically stable if and only if all eigenvalues of $J_f(x_0)$ have negative real part, so let us use this condition instead of asymptotic stability in the proof. Suppose all eigenvalues of $J_f(x)$ have negative real part. Two of these are the real negative $-d_S$ and $-d_E$, whose product is $d_S d_E$. Since the coefficients of $J_f(x)$ are real, its characteristic polynomial has real coefficients, and so its complex eigenvalues come in conjugate pairs. So, $J_f(x)$ has a further eigenvalue z with negative real part and non-zero imaginary part, then $\bar{z}$ is the fourth eigenvalue, and $\det J_f(x) = d_S d_E z\bar{z} = d_S d_E |z|^2 > 0$. Otherwise, all eigenvalues of $J_f(x)$ are real and negative, and thus $\det J_f(x)$ is the product of four negative real numbers and again is positive.
Conversely, suppose $\det J_f(x) > 0$. We argue similarly: two eigenvalues are negative and real, $-d_S$ and $-d_E$. The other two must then either be a pair of complex conjugates, in which case their real parts are the same and must be negative because $J_f(x)$ has at least three eigenvalues with negative real part, or must be real numbers, at least three of which are negative, and then the fourth must be negative as well since their product is $\det J_f(x) > 0$. □

Theorem 2.

Proof. We will go back to discussing the J_f in terms of partial derivatives of f rather than explicit values here. By expanding its determinant by minors first across the row of $-d_S$ and then down the column of $-d_E$, we obtain

$$\det J_f = d_S d_E \begin{vmatrix} \frac{\partial f_Z}{\partial Z} & \frac{\partial f_Z}{\partial M} \\ \frac{\partial f_M}{\partial Z} & \frac{\partial f_M}{\partial M} \end{vmatrix}.$$

Call the matrix whose determinant appears on the right A_f. Then, since $d_S d_E > 0$, $\det A$ and $\det J_f$ have the same sign, so by the lemma it follows that an equilibrium is stable if and only if $\det A_f > 0$ at the equilibrium. Now, a fixed point of $F \circ G$ is asymptotically stable if and only if the derivative $(F \circ G)'(Z) = F'(G(Z))G'(Z) = F'(M)G'(Z)$ is less than one in magnitude. Since $F \circ G$ is increasing, its derivative is always positive, so we can safely ignore the condition of magnitude and take this condition to be that $F'(M)G'(Z) < 1$. Thus, the statement of the theorem is equivalent to showing that $\det A$ and $1 - F'(M)G'(Z)$ have the same sign. Let us write the component functions f_Z and f_M of the function f in terms of the functions F and G,

$$f_Z = (F(M) - Z)(k_{MZ} M + d_Z),$$
$$f_M = (G(Z) - M)(k_{MZ} Z + d_M)$$

whereby in terms of these functions, we have

$$\frac{\partial f_Z}{\partial M} = F'(M)(k_{MZ} M + d_Z) + k_{MZ}(F(M) - Z),$$
$$\frac{\partial f_M}{\partial Z} = G'(Z)(k_{MZ} Z + d_M) + k_{MZ}(G(Z) - M).$$

But, at an equilibrium point $F(M) = Z$ and $G(Z) = M$, so the second term is zero in each expression and we have simply

$$\frac{\partial f_Z}{\partial M} = F'(M)(k_{MZ}M + d_Z),$$
$$\frac{\partial f_M}{\partial Z} = G'(Z)(k_{MZ}Z + d_M)$$

and also

$$\frac{\partial f_Z}{\partial Z} = -(k_{MZ}M + d_Z),$$
$$\frac{\partial f_M}{\partial M} = -(k_{MZ}Z + d_M).$$

So, the determinant of A, using the expression in terms of these functions, is

$$\begin{aligned}\det(A) &= \frac{\partial f_Z}{\partial Z} \cdot \frac{\partial f_M}{\partial M} - \frac{\partial f_Z}{\partial M} \cdot \frac{\partial f_M}{\partial Z} \\ &= (k_{MZ}M + d_Z)(k_{MZ}Z + d_M) - F'(M)G'(Z)(k_{MZ}M + d_Z)(k_{MZ}Z + d_M) \\ &= (k_{MZ}M + d_Z)(k_{MZ}Z + d_M)(1 - F'(M)G'(Z)).\end{aligned}$$

Since $k_{MZ}M + d_Z$ and $k_{MZ}Z + d_M$ are both positive, this has the same sign as $1 - F'(M)G'(Z)$, which is the necessary result. ☐

Lemma 2.

Proof. This can seen through linearization about $X = (S, M, Z, E) \in \mathbb{R}^4_+$. The Jacobian is

$$\begin{pmatrix} -d_S & 0 & 0 & 0 \\ \frac{K_S k_Z}{(K_S+S)^2} & -d_Z - k_{MZ}M & -k_{MZ}Z & 0 \\ -\frac{k_M K_S K_Z^3}{(K_Z+Z)(K_Z S+K_S(K_Z+Z))^2} & -k_{MZ}M - \frac{k_M K_S K_Z^2(K_Z S+2K_S(K_Z+Z))}{(K_Z+Z)^2(K_Z S+K_S(K_Z+Z))^2} & -d_M - k_{MZ}Z & 0 \\ 0 & -\frac{k_E K_Z}{(K_Z+Z)^2} & 0 & -d_E \end{pmatrix}$$

and we see that two eigenvalues have negative real parts, $-d_E$ and $-d_S$.
A third eigenvalue also have negative real parts, and the fourth is negative whenever $Z = 0$ or whenever $Z > 0$ and

$$\begin{aligned}0 < k_M <&C(\theta^*, S, Z, M) \\ &\frac{(K_Z + Z)^2(d_M(d_Z + k_{MZ}M) + d_Z k_{MZ}Z)(K_Z S + K_S(K_Z + Z))^2}{k_{MZ}K_S K_Z^2 Z(K_Z S + 2K_S(K_Z + Z))}\end{aligned}$$

☐

Proposition 1.

Proof. Recall at equilibrium we have

$$Z = F(M) = \frac{k_Z}{d_Z + k_{MZ}M} \cdot \frac{S}{K_S + S}.$$

Solving for M, we have

$$M = \frac{1}{k_{MZ}} \left(\frac{k_Z}{Z} \cdot \frac{S}{K_S + S} - d_Z \right).$$

At the same time, at equilibrium, we have

$$M = G(Z) = \frac{k_M}{d_M + k_{MZ} Z} \cdot \frac{K_Z}{K_Z + Z} \cdot \frac{1}{1 + \frac{Z}{K_Z} + \frac{S}{K_S}}.$$

Equating these these expressions for M, we see that the equilibrium values of Z satisfy

$$\frac{1}{k_{MZ}} \left(\frac{k_Z}{Z} \cdot \frac{S}{K_S + S} - d_Z \right) = \frac{k_M}{d_M + k_{MZ} Z} \cdot \frac{K_Z}{K_Z + Z} \cdot \frac{1}{1 + \frac{Z}{K_Z} + \frac{S}{K_S}}.$$

Taking the reciprocal on each side, we have

$$k_{MZ} \left(\frac{1}{\frac{k_Z}{Z} \cdot \frac{S}{K_S+S} - d_Z} \right) = \left(\frac{k_{MZ}}{k_M} Z + \frac{d_M}{k_M} \right) \left(1 + \frac{Z}{K_Z} \right) \left(1 + \frac{Z}{K_Z} + \frac{S}{K_S} \right).$$

Inserting $M_{\text{max}} = \frac{k_M}{d_M}$ and $Z_{\text{max}} = \frac{k_Z}{d_Z}$ and factoring k_Z from the left-hand side, we have

$$\frac{k_{MZ}}{k_Z} \left(\frac{1}{\frac{1}{Z} \cdot \frac{S}{K_S+S} - \frac{1}{Z_{\text{max}}}} \right) = \left(\frac{k_{MZ}}{k_M} Z + \frac{1}{M_{\text{max}}} \right) \left(1 + \frac{Z}{K_Z} \right) \left(1 + \frac{Z}{K_Z} + \frac{S}{K_S} \right).$$

Finally, we multiply the second fraction of the left-hand side by Z and clear its denominator, giving

$$\frac{k_{MZ}}{k_Z} Z = \left(\frac{k_{MZ}}{k_M} Z + \frac{1}{M_{\text{max}}} \right) \left(1 + \frac{Z}{K_Z} \right) \left(1 + \frac{Z}{K_Z} + \frac{S}{K_S} \right) \left(\frac{S}{K_S + S} - \frac{Z}{Z_{\text{max}}} \right)$$

□

Proposition 2.

Proof. Recall the polynomial relation which gives the equilibrium values of Z:

$$\frac{k_{MZ}}{k_Z} Z = \left(\frac{k_{MZ}}{k_M} Z + \frac{1}{M_{\text{max}}} \right) \left(1 + \frac{Z}{K_Z} \right) \left(1 + \frac{Z}{K_Z} + \frac{S}{K_S} \right) \left(\frac{S}{K_S + S} - \frac{Z}{Z_{\text{max}}} \right).$$

For $Z > 0$, the left-hand side is positive, so the right-hand side must be positive. The first three terms of the right-hand side are automatically positive, and the last term must also be positive at all such values. The function $\frac{S}{K_S+S} = \frac{1}{1+K_S/S}$ is strictly increasing in S for S positive. For a given fixed biologically reasonable Z, the right-hand side is strictly increasing in S for $S > 0$, and the result follows. □

Theorem 3.

Proof. We multiply the polynomial relation of Z from Proposition 1 by $K_S + S$, giving

$$\frac{k_{MZ}}{k_Z} Z(K_S + S) = \left(\frac{k_{MZ}}{k_M} Z + \frac{1}{M_{\text{max}}} \right) \left(1 + \frac{Z}{K_Z} \right) \left(1 + \frac{Z}{K_Z} + \frac{S}{K_S} \right) \left(S - \frac{Z}{Z_{\text{max}}} (K_S + S) \right),$$

a quadratic relation in S. Expanding to form $aS^2 + bS + c = 0$ using Mathematica, we attain

$$\begin{aligned}
a &= \frac{1}{K_S}\left(\frac{1}{M_{\text{max}}} + \frac{k_{MZ}}{k_M}Z\right)\left(1 + \frac{Z}{K_Z}\right)\left(1 - \frac{Z}{Z_{\text{max}}}\right) \\
b &= -\frac{k_{MZ}}{k_Z}Z + \left(\frac{1}{Z_{\text{max}}} + \frac{k_{MZ}}{k_M}Z\right)\left(1 + \frac{Z}{K_Z}\right)\left(1 + \frac{Z}{K_Z} - \frac{2Z}{Z_{\text{max}}} - \frac{Z^2}{K_Z Z_{\text{max}}}\right) \\
c &= -K_S Z\left(\frac{k_{MZ}}{k_Z} + \frac{1}{Z_{\text{max}}}\left(\frac{1}{M_{\text{max}}} + \frac{k_{MZ}}{k_M}Z\right)\left(1 + \frac{Z}{K_Z}\right)^2\right).
\end{aligned}$$

Putting

$$\begin{aligned}
v &= \frac{1}{M_{\text{max}}} + \frac{k_{MZ}}{k_M}Z \\
w &= 1 + \frac{Z}{K_Z} \\
x &= 1 - \frac{Z}{Z_{\text{max}}} \\
y &= \frac{k_{MZ}}{k_Z}Z
\end{aligned}$$

we simplify the coefficients to

$$\begin{aligned}
a &= \frac{1}{K_S}vwx \\
b &= -y + vw(wx + x - 1) \\
c &= -K_S(y + vw^2(1 - x))
\end{aligned}$$

Dividing through by vwx, we attain an equation of the form $a'S^2 + b'S + c' = 0$ where

$$\begin{aligned}
a' &= \frac{1}{K_S} \\
b' &= -\frac{y}{vwx} + w + 1 - \frac{1}{x} \\
c' &= -K_S\left(\frac{y}{vwx} - w + \frac{w}{x}\right).
\end{aligned}$$

Noting

$$a'c' = -\frac{y}{vwx} + w + \frac{w}{x} = b' - 1 + \frac{1}{x} - \frac{w}{x} = b' - \frac{w + x - 1}{x},$$

the discriminant d is given by

$$d = b'^2 - 4a'c' = b'^2 - 4b' + 4\frac{w + x - 1}{x},$$

and by completing the square we have

$$d = (b' - 2)^2 + 4\frac{w - 1}{x}.$$

Since $Z \in (0, Z_{\text{max}})$, we have $w > 1$ and $x > 0$ and thus $d > 0$, whereby the quadratic equation in S will always have two distinct solutions. The product of these solutions is equal to $\frac{c'}{a'}$, which can be written as

$$\frac{c'}{a'} = -K_S^2\left(\frac{y}{vwx} + w\left(\frac{1}{x} - 1\right)\right).$$

Observe that $v, w, x, y > 0$ and $0 < x < 1$. Therefore, $\frac{1}{x} > 1$. Hence, the entire inner term is positive and $\frac{c'}{a'}$ is negative. Thus, of the two distinct real solutions, one must be positive and one must be negative. Thus, there exists a positive solution S for any $Z \in (0, Z_{\text{max}})$. $\square$

4. SUMMARY RESTATEMENT OF MATHEMATICAL MODELS AND PARAMETERS

Supplementary Table 7. Structure of an ODE-based (Lipschitz) mathematical model for (non-negative) expression of gene "G"

$$\frac{d[G]}{dt} = B_G + k_{a_G} \prod_{i=1}^{n_G} \sum_{j_i=1}^{m_{G,i}} \left(\frac{[TF_{j_i}]/K_{ij_i}}{1 + \sum_{k_i=1}^{m_{G,i}} [TF_{k_i}]/K_{ik_i}} \right)$$

$$+ k_{r_G} \prod_{u=1}^{s_G} \sum_{v_u=1}^{q_{G,u}} \left(\frac{1}{1 + \sum_{w_u=1}^{q_{G,u}} [TF_{w_u}]/K_{uw_u}} \right) - d_G[G]$$

The mathematical model used for transcriptional regulation is an ordinary differential equation, using Michaelis-Menten reaction kinetics.

Variables are:

G: Gene

TF: Transcription Factor

Initial conditions:

[G] ∈ $[0, a]$; $[TF_i] \in [0, a_i]$ for every $i \in \{1, \ldots, m_G\}$

Notes:

The model is for (non-negative) gene expression. For transcriptional activation and repression, we prescribe Michaelis-Menten-style kinetics. This regulatory structure is based on the following assumptions: (i) transcriptional activation is proportional to the number of binding domains occupied by transcriptional activators and (ii) transcriptional repression is proportional to the number of binding domains occupied by transcriptional repressors.

Underlying reactions for these ODEs are defined in Supplementary Table 8.
Parameters are defined in Supplementary Table 9.

Supplementary Table 8. Reaction kinetics for model of Supplementary Table 7

Reaction	Kinetics	Description and Notes
$* \longrightarrow G$	B_G	Baseline synthesis
$* \xrightarrow{Act.Dom.\text{ of G}} [A_i: i = 1, \dots, n_G]$	$\left[A_i \stackrel{\text{def}}{=} \sum_{j_i=1}^{m_{G,i}} \left(\frac{[TF_{j_i}]/K_{ij_i}}{1 + \sum_{k_i=1}^{m_{G,i}} [TF_{k_i}]/K_{ik_i}} \right) : i = 1, \dots, n_G \right]$	Additive activities of competitive activators of domains
$[A_i: i = 1, \dots, n_G] \xrightarrow{Coop.Act.} G$	$k_{a_G} \prod_{i=1}^{n_G} A_i$	Cooperative activities of domain activations
$* \xrightarrow{Rep.Dom.\text{ of G}} [R_u: u = 1, \dots, S_G]$	$\left[R_u \stackrel{\text{def}}{=} \sum_{v_u=1}^{q_{G,u}} \left(\frac{1}{1 + \sum_{w_u=1}^{q_{G,u}} [TF_{w_u}]/K_{uw_u}} \right) : u = 1, \dots, s_G \right]$	Additive activities of competitive repressors of domains
$[R_u: u = 1, \dots, s_G] \xrightarrow{Coop.Rep.} G$	$k_{r_G} \prod_{u=1}^{s_G} R_u$	Cooperative activities of domain repressors
$G \longrightarrow *$	$d_G[G]$	G decay

Supplementary Table 9. Parameters for the model of Supplementary Table 7.

Parameter	Description
k_{a_G}	Activation rate of *G*
k_{r_G}	Repression rate of *G*
K_{ij_i}	Activation constant of binding domain type *i* by transcriptional activator j_i for *G*
K_{uw_u}	Repressor constant of binding domain type *u* by transcriptional repressor w_u for *G*
B_G	Baseline constant (constitutive) production of *G*
d_G	Decay of *G*

Supplementary Table 10. Core model for TGF-b induced EMT

$\frac{d[T]}{dt} = 0$
$\frac{d[Z]}{dt} = \frac{k_Z[T]}{K_T + [T]} - k_{ZM}[Z][M] - d_Z[Z]$
$\frac{d[M]}{dt} = k_M \left(\frac{1}{1 + \frac{[Z]}{K_{Z'}}} \right) \left(\frac{1}{1 + \frac{[Z]}{K_{Z'}} + \frac{[T]}{K_T}} \right) - k_{ZM}[Z][M] - d_M[M]$
$\frac{d[E]}{dt} = \frac{k_E K_{Z''}}{K_{Z''} + [Z]} - d_E[E]$

Variables are:

T: TGFb

Z: ZEB1/2

M: MicroRNA-200

E: E-cadherin / CDH1

Initial condition:

[Z] = 0; [M] = [E] = 1; [T] ∈ $[0, a]$

Underlying reactions for these ODEs are defined in Supplementary Table 11. Note the appearance of a negative quadratic complexing term, whose inclusion does not alter the existence of bounded solutions for this model. Parameters are defined in Supplementary Table 12.

Supplementary Table 11. Reaction kinetics for the core model of Supplementary Table 10.

**Reaction**	**Kinetics**	**Description and Notes**
$* \xrightarrow{T} Z$	$\frac{k_Z[T]}{K_T + [T]}$	Activation of *Z* by *T*
$* \xrightarrow{Z} E$	$\frac{k_E K_{Z''}}{K_{Z''} + [Z]}$	Repression of *E* by *Z*
$Z, M \longrightarrow *$	$k_{ZM}[Z][M]$	Complexing of *M* and *Z*

$* \xrightarrow{T,Z} M$	$k_M R_{Zbox} R_{Ebox}$ $R_{Zbox} \stackrel{\text{def}}{=} \frac{1}{1 + \frac{[Z]}{K_{Z'}}}$ $R_{Ebox} \stackrel{\text{def}}{=} \frac{1}{1 + \frac{[Z]}{K_{Z'}} + \frac{[T]}{K_T}}$	Cooperative repression of *M* by (i) Z-box activity by *Z* (R_{Zbox}) and (ii) E-box activities by *Z* and *T* (R_{Ebox})
$Z \longrightarrow *$	$d_Z[Z]$	Decay of *Z*
$M \longrightarrow *$	$d_M[M]$	Decay of *M*
$E \longrightarrow *$	$d_E[E]$	Decay of *E*

Supplementary Table 12. Parameters for the core model of Supplementary Table 10.

Parameter	Representative Value	Description
k_Z	0.1	Activation/repression rate of *Z*
k_M	1	Activation/repression rate of *M*
k_E	1	Activation/repression rate of *E*
k_{ZM}	100	Complexing rate of *M* and *Z*
K_T	1	Activation/repression constant of *T*
$K_{Z'}$	0.1	Activation/repression constant of *Z* on *M*
$K_{Z''}$	0.1	Activation/repression constant of *Z* on *E*
d_Z	k_Z	Decay rate of *Z* ; constrained by unit bound (see note)
d_M	k_M	Decay rate of *M* ; constrained by unit bound (see note)
d_E	k_E	Decay rate of *E* ; constrained by unit bound (see note)

Note: The steady-state values of *Z*, *M,* and *E* for the model in Supplementary Table 11 are respectively bounded above by k_Z/d_Z, k_M/d_M, and k_E/d_E. We equate these to unity ("unit bounds").

Exhibits for the Core System

In Supplementary Tables 13a and 13b, the parameter $K_{Z'}$ is varied and the remaining parameters are fixed. When $K_{Z'} = 0.1$, the system admits a hysteresis bifurcation. When $K_{Z'} = 1.0$, the hysteresis bifurcation effectively vanishes. Furthermore, the parameters (k_Z, $K_{Z'}$, k_{MZ})

partition their spaces into hysteresis and non-hysteresis subspaces, while the remaining parameters do not admit such partitions. Hence non-separable parameters are free parameters and may be set to any value no matter how small or large without changing any qualitative behaviors or conclusions. Therefore, the parameters (K_T, k_M, k_E) are set to ``unit'' values.

Supplementary Table 13a. Representative parameter values for "hysteresis" for the model of Supplementary Table 10.

Parameter	Value
k_Z	0.1
k_M	1
k_E	1
k_{MZ}	100
K_T	1
$K_{Z'}$	0.1
$K_{Z''}$	0.1
d_Z	k_Z
d_M	k_M
d_E	k_E

Supplementary Table 13b. Representative parameter values of "no hysteresis" for the model of Supplementary Table 10.

Parameter	Value
k_Z	0.1
k_M	1
k_E	1
k_{MZ}	100
K_T	1
$K_{Z'}$	1.0
$K_{Z''}$	0.1
d_Z	k_Z

d_M	k_M
d_E	k_E

Supplementary Table 14. Analysis of core model of Supplementary Table 10 by High Dimensional Model Representation (HDMR); "Global Sensitivity Analysis"

Symbol	Definition	Description and Notes
X_0	$\{x_{0a}, x_{0b}\}$; $x_{0a} = (0,1,1)$; $x_{0b} = (1,0,0)$	Initial conditions for *X=(Z,M,E)*
θ	$\theta = (\theta_1, \dots, \theta_n)$; $\theta_{-i} = (\theta_1, \dots, \theta_{i-1}, \theta_{i+1} \dots, \theta_n)$;	Model parameter vector; Notation for implicit vector
μ	Independent uniform	Probability measure on Θ
$f(\theta)$	$\int_0^{T_{max}} \left(E_{\infty b}(t,\theta) - E_{\infty a}(t,\theta)\right) \; dt$	Hysteresis area; $E_{\infty b}(t,\theta)$ is the equilibrium of *E* for $T_0 = t$ and initial condition x_{0b} and $E_{\infty a}(t,\theta)$ similarly with x_{0b} ; *X* (and hence *E*) is a function the model parameters (θ)
f_0	$\int_\Theta f(\theta) d\nu(\theta)$	HDMR constant component function; mean hysteresis area
$f_i(\theta_i)$	$\int_{\Theta_{-i}} f(\theta_{-i}, \theta_i) d\nu_{-i}(\theta_{-i}) - f_0$	HMDR first-order (univariate) component function in θ_i
$f_{ij}(\theta_i, \theta_j)$	$\int_{\Theta_{-ij}} f(\theta_{-ij}, \theta_i, \theta_j) d\nu_{-ij}(\theta_{-ij}) - f_i(\theta_i) - f_j(\theta_j) - f_0$	HMDR second-order (univariate) component function in θ_i and θ_j
$\mathbb{V}$	Var(f)	Variance of *f* with respect to μ on Θ
$\mathbb{S}_i$	$\mathbb{V}^{-1} \int_{\Theta_i} f_i^2(\theta_i) d\nu_i(\theta_i)$	First-order sensitivity index of parameter θ_i
$\mathbb{S}_{ij}$	$\mathbb{V}^{-1} \int_{\Theta_{ij}} f_{ij}^2(\theta_i) d\nu_{ij}(\theta_i, \theta_j)$	Second-order sensitivity index of parameter vector $\theta_{ij} = (\theta_i, \theta_j)$

Random sampling high dimensional model representation (RS-HDMR) is used to examine the contributions of model parameters to hysteresis area. Independent and identically distributed random samples are attained, and RS-HDMR used to estimate the component functions and sensitivity indices to second-order.

Supplementary Table 15. Structure of an ODE-based (Lipschitz) mathematical model for (non-negative) autocrine regulation of TGFb (*T*); ("autocrine model")

$\frac{d[T_f]}{dt} = \frac{k_a[T_b]}{K_{a+}[T_b]} - \frac{k_b[T_f]}{K_{b+}[T_f]} - d_{T_f}[T_f]$
$\frac{d[T_b]}{dt} = \frac{k_b[T_f]}{K_{b+}[T_f]} - d_{T_b}[T_b]$

Variables are:

T_f free TGFb ("unbound")

T_b bound TGFb ("bound")

Initial condition:

$[T_f]$ = 1; $[T_b]$ = 0

Underlying reactions for these ODEs are defined in Supplementary Table 16.
Parameters are defined in Supplementary Table 17.

Note: the functional form of the general model is elected for representation of autocrine signaling, conferring similar timescales of the autocrine and "core" models.

Supplementary Table 16. Reaction kinetics for the autocrine model of Supplementary Table 15.

Reaction	Kinetics	Description and Notes
$* \xrightarrow{T_b} T_f$	$\frac{k_a[T_b]}{K_a + [T_b]}$	Activation of T_f by T_b (stimulated production)
$* \xrightarrow{T_f} T_b$<br>$T_f \longrightarrow *$	$\frac{k_b[T_f]}{K_{b+}[T_f]}$	Activation of T_b by T_f (induced signaling)
$T_f \longrightarrow *$	$d_{T_f}[T_f]$	Decay of T_f
$T_b \longrightarrow *$	$d_{T_b}[T_b]$	Decay of T_b

Supplementary Table 17. Parameters for the autocrine model of Supplementary Table 15.

**Parameter**	**Representative Value**	**Description**
k_a	1	Activation rate of T_f (autocrine)
k_b	1	Activation rate of T_b (induced signaling)
K_a	1	Activation constant of T_f (autocrine)
K_b	1	Activation constant of T_b (induced signaling)
d_{T_f}	1	Decay rate of T_f
d_{T_b}	1	Decay rate of T_b

Supplementary Table 18. Structure of an ODE-PDE mathematical model for (non-negative); "paracrine model"

$\frac{d[T_f]}{dt} = c\Delta[T_f] + \delta_A\left(\frac{k_a[T_b]}{K_{a+}[T_b]} - \frac{k_b[T_f]}{K_{b+}[T_f]}\right) - d_{T_f}[T_f]$
$\frac{d[T_b]}{dt}$ as in Supplementary Table 15; $\frac{d[Z]}{dt}$, $\frac{d[M]}{dt}$, and $\frac{d[E]}{dt}$ as in Supplementary Table 10.

Note: the variable T_f has spatial dependence through diffusion and point source configuration of the cellular field (at discrete points). The set $A \subset \{(x, y): (x, y) \in \mathbb{R}^2\}$ is a collection of discrete points, indicating the locations of cells. All variables are described in Tables S4 and S11.

This model is spatially discretized on an equispaced *k* x *k* grid, where T_f experiences spatial dynamics (diffusion). Using a second-order finite difference scheme on the discretized domain, the *method of lines* is used to express the ODE-PDE model as a (large) system of ODEs, which can then be efficiently numerically integrated.

Initial condition:

$$[T_f] = [d_{ij}: (i, j) \in \{1, \dots, k\}^2]$$

Other initial conditions as described in Tables S5 and S6.

Underlying reactions for these ODEs are defined in Supplementary Table 19.

Parameters are defined in Supplementary Table 20.

Supplementary Table 19. Reaction kinetics for the paracrine model of Supplementary Table 18.

**Reaction**	**Kinetics**	**Description and Notes**
$* \xrightarrow{T_b} T_f$ and $T_f \longrightarrow *$	$\delta_A\left(\frac{k_a[T_b]}{K_{a+}[T_b]} - \frac{k_b[T_f]}{K_{b+}[T_f]}\right)$	Net activation or suppression of T_f at the points $A \subset \{(x,y):(x,y) \in \mathbb{R}^2\}$, each having cellular dynamics described the autocrine and core models.
$T_f \longrightarrow T_f$	$c\Delta[T_f]$	Diffusion of T_f

Supplementary Table 20. Parameters for the paracrine model of Supplementary Table 18.

**Parameter**	**Representative Value**	**Description**
k	51	Number of grid elements in one dimension; defines the *k* x *k* grid
c	10^{-4}	Diffusion coefficient
ΔA	10^{-2}	Distance between adjacent (vertical or horizontal) locations on the grid

References

[1] Benjamin Cieply, Philip Riley, Phillip M. Pifer, Joseph Widmeyer, Joseph B. Addison, Alexey V. Ivanov, James Denvir, and Steven M. Frisch. Suppression of the epithelial–mesenchymal transition by grainyhead-like-2. *Cancer Research*, 72(9):2440, 05 2012.

[2] Philip A Gregory, Cameron P Bracken, Eric Smith, Andrew G Bert, Josephine A Wright, Suraya Roslan, Melanie Morris, Leila Wyatt, Gelareh Farshid, Yat-Yuen Lim, Geoffrey J Lindeman, M Frances Shannon, Paul A Drew, Yeesim Khew-Goodall, and Gregory J Goodall. An autocrine tgf-/zeb/mir-200 signaling network regulates establishment and maintenance of epithelial-mesenchymal transition. *Molecular Biology of the Cell*, 22(10):1686–1698, 05 2011.

[3] Joan Massagué and David Wotton. New embo member's review: Transcriptional control by the tgf-/smad signaling system. *The EMBO Journal*, 19(8):1745–1754, 04 2000.

[4] C. P. Bracken, P. A. Gregory, N. Kolesnikoff, A. G. Bert, J. Wang, M. F. Shannon, and G. J. Goodall. A double-negative feedback loop between zeb1-sip1 and the microrna-200 family regulates epithelial-mesenchymal transition. *Cancer Res*, 68(19):7846–54, 2008.

[5] S. Brabletz and T. Brabletz. The zeb/mir-200 feedback loop–a motor of cellular plasticity in development and cancer? *EMBO Rep*, 11(9):670–7, 2010.

[6] Sun-Mi Park, Arti B Gaur, Ernst Lengyel, and Marcus E Peter. The mir-200 family determines the epithelial phenotype of cancer cells by targeting the e-cadherin repressors zeb1 and zeb2. *Genes & Development*, 22(7):894–907, 04 2008.

[7] U. Burk, J. Schubert, U. Wellner, O. Schmalhofer, E. Vincan, S. Spaderna, and T. Brabletz. A reciprocal repression between zeb1 and members of the mir-200 family promotes emt and invasion in cancer cells. *EMBO Rep*, 9(6):582–9, 2008.

[8] John Guckenheimer and Philip Holmes. *Nonlinear oscillations, dynamical systems, and bifurcations of vector fields*. Applied mathematical sciences. Springer, New York, 2002.

[9] William E. Schiesser. *The Numerical Method of Lines: Integration of Partial Differential Equations*. Academic Press, 1991.

[10] Herschel Rabitz and Ömer F. Alış. General foundations of highdimensional model representations. *Journal of Mathematical Chemistry*, 25(2-3):197–233, 1999.

[11] Erhan Cinlar. *Probability and Stochastics*. Springer-Verlag New York Springer-Verlag New York, 2011.

[12] Giles Hooker. Generalized functional anova diagnostics for high-dimensional functions of dependent variables. *Journal of Computational and Graphical Statistics*, 16(3):709–732, 2007.

[13] I. M. Sobol. On sensitivity estimation for nonlinear mathematical models. *Matem. Mod.*, 2(1):112–118, 1990.

[14] I.M. Sobol. Global sensitivity indices for nonlinear mathematical models and their monte carlo estimates. *Mathematics and Computers in Simulation*, 55(1-3):271–280, 2001.

[15] Andrea Saltelli. Making best use of model evaluations to compute sensitivity indices. *Computer Physics Communications*, 145(2):280–297, 2002.

[16] Andrea Saltelli, Paola Annoni, Ivano Azzini, Francesca Campolongo, Marco Ratto, and Stefano Tarantola. Variance based sensitivity analysis of model output. design and estimator for the total sensitivity index. *Computer Physics Communications*, 181(2):259–270, 2010.

[17] G.G. Lorentz, M.V. Golitschek, and Y. Makovoz. *Constructive Approximation*. Spring, New York, 1996.

[18] Ronald Aylmer Fisher. On the ”probable error” of a coefficient of correlation deduced from a small sample. *Metron*, 1(4):3–32, 1921.

[19] Wassily Hoeffding. A class of statistics with asymptotically normal distribution. *Ann. Math. Statist.*, 19(3):293–325, 09 1948.

[20] Jaroslav Hajek. Asymptotic normality of simple linear rank statistics under alternatives. *Ann. Math. Statist.*, 39(2):325–346, 04 1968.

[21] Genyuan Li and Herschel Rabitz. General formulation of hdmr component functions with independent and correlated variables. *Journal of Mathematical Chemistry*, 50(1):99–130, 2012.

[22] Grzegorz A. Rempała and Jacek Wesołowski. Double asymptotics for the chi-square statistic. *Statistics & Probability Letters*, 119:317–325, 12 2016.

[23] Gaelle Chastaing, Fabrice Gamboa, and Clémentine Prieur. Generalized hoeffding-sobol decomposition for dependent variables - application to sensitivity analysis. *Electron. J. Statist.*, 6:2420–2448, 2012.

[24] Sanjay R. Arwade, Mohammadreza Moradi, and Arghavan Louhghalam. Variance decomposition and global sensitivity for structural systems. *Engineering Structures*, 32(1):1 – 10, 2010.

[25] Akimichi Takemura. Tensor analysis of anova decomposition. 78(384):894–900, 1983.

[26] Ömer F. Alış and Herschel Rabitz. Efficient implementation of high dimensional model representations. *Journal of Mathematical Chemistry*, 29(2):127–142, 2001.

[27] Genyuan Li, Jishan Hu, Sheng-Wei Wang, Panos G. Georgopoulos, Jacqueline Schoendorf, and Herschel Rabitz. Random sampling-high dimensional model representation (rs-hdmr) and orthogonality of its different order component functions. *The Journal of Physical Chemistry A*, 110(7):2474–2485, 2006. PMID: 16480307.
